# Two – three loci control scleral ossicle formation via epistasis in the cavefish *Astyanax mexicanus*

**DOI:** 10.1371/journal.pone.0171061

**Published:** 2017-02-09

**Authors:** Anastasia Lyon, Amanda K. Powers, Joshua B. Gross, Kelly E. O’Quin

**Affiliations:** 1 Biology Program, Centre College, Danville, KY, United States of America; 2 Department of Biological Sciences, University of Cincinnati, Cincinnati, OH, United States of America; Xiamen University, CHINA

## Abstract

The sclera is the protective outer layer of the eye. In fishes, birds, and reptiles, the sclera may be reinforced with additional bony elements called scleral ossicles. Teleost fish vary in the number and size of scleral ossicles; however, the genetic mechanisms responsible for this variation remain poorly understood. In this study, we examine the inheritance of scleral ossicles in the Mexican tetra, *Astyanax mexicanus*, which exhibits both a cave morph and a surface fish morph. As these morphs and their hybrids collectively exhibit zero, one, and two scleral ossicles, they represent a microcosm of teleost scleral ossicle diversity. Our previous research in F2 hybrids of cavefish from Pachón cave and surface fish from Texas suggested that three genes likely influence the formation of scleral ossicles in this group through an epistatic threshold model of inheritance, though our sample size was small. In this study, we expand our sample size using additional hybrids of Pachón cavefish and Mexican surface fish to (1) confirm the threshold model of inheritance, (2) refine the number of genes responsible for scleral ossicle formation, and (3) increase our power to detect quantitative trait loci (QTL) for this trait. To answer these three questions, we scored surface fish and cavefish F2 hybrids for the presence of zero, one, or two scleral ossicles. We then analyzed their distribution among the F2 hybrids using a chi-square (χ^2^) test, and used a genetic linkage map of over 100 microsatellite markers to identify QTL responsible for scleral ossicle number. We found that inheritance of scleral ossicles follows an epistatic threshold model of inheritance controlled by two genes, which contrasts the three-locus model estimated from our previous study. Finally, the combined analysis of hybrids from both crosses identified two strong QTL for scleral ossicle number on linkage groups 4.2 and 21, and a weaker QTL on linkage group 4.1. Scleral ossification remains a complex trait with limited knowledge of its genetic basis. This study provides new insight into the number and location of genes controlling the formation of scleral ossicles in a teleost fish species.

## Introduction

### Vertebrate sclera and scleral ossicles

The sclera is the protective layer of collagen and elastic fiber surrounding the eye. In mammals, the sclera is a flexible, fibrous tissue; but in fishes, birds, and reptiles, the sclera may be reinforced with an additional ring of cartilage or even small bones. These bony elements, known as scleral ossicles, provide additional support and may even serve as sites of muscle attachment [1]. The formation of scleral ossicles has been studied most extensively in chicks (reviewed in [2]). In this system, the scleral ossicles are derived from neural crest cells that are induced by scleral papillae to form a ring of 8–15 bones via intramembranous ossification [2]. Although the genetic factors responsible for the induction and ossification of chick scleral ossicles remain unknown, Bone morphogenetic protein (*bmp*), Sonic hedgehog (*shh)*, and Fibroblast growth factor (*fgf)* have all been implicated [3–5].

More recent research has focused on the evolution and development of scleral ossicles in teleost fish, which comprise one of the largest and most diverse subsets of vertebrates on earth (reviewed in [6]). Similar to chickens, the scleral ossicles of teleost fish develop from neural crest cells [7]. Unlike chicks, however, the scleral ossicles of teleosts are not induced by papillae, form only one or two ossicles, and ossify via endochondral ossification [6,8]. These differences raise the possibility that the scleral ossicles of fish are not homologous to the scleral ossicles of birds and other reptiles [8]. Given their vast diversity in size, shape, and habitat, it may be unsurprising that teleosts exhibit considerable variation in the number and size of scleral ossicles [6,9]. Roughly half of teleost species so far examined lack scleral ossicles entirely, while most of the remaining species form only two ossicles that may or may not encircle the entire circumference of the eye [6,9]. The genetic, developmental, and evolutionary causes of this diversity are currently unknown, although it may be tied to evolution within deep-sea and benthic environments, since teleosts found in these habitats tend to have fewer scleral ossicles than do teleosts from other habitats [6]. In any case, what is clear is that this diversity appears to be the result of numerous independent gains and losses [6].

### The *Astyanax mexicanus* model system

*Astyanax mexicanus*, more commonly known as the Mexican tetra, is a teleost fish found throughout Mexico and Texas [10]. These fish exhibit two morphs: an eyed surface fish (SF) form and a blind cave fish (CF) form. *Astyanax* SF, like other Characiform fishes, possess two elongated scleral ossicles surrounding each eye [8]. Starting from the anterior and posterior ends of a cartilaginous scleral ring, these ossicles elongate over a period of two years via unilateral perichondrial ossification, until they fuse into a single bony ring surrounding almost the entire perimeter of the eye [8]. In *Astyanax* CF, however, this process is altered. Although CF do form a cartilaginous scleral ring, this ring does not ossify, and thus the scleral ossicles never form [11]. Eventually, as the eye stops growing, the cartilaginous scleral ring forms a cyst around the degenerating CF eye [11]. Currently, there are approximately 30 known populations of *Astyanax* CF [10]. Some of these CF populations actually retain the wild-type scleral ossification observed in SF, including the Río Subterraneo and Tinaja populations [12–13]. However, most CF populations so far sampled do not retain scleral ossicles, and scleral ossification has likely been lost independently multiple times in this group [13]. Thus, *Astyanax* exhibit microevolutionary patterns of scleral ossicle evolution that mirror the larger macroevolutionary patterns observed in teleosts more generally. Combined with the fact that *Astyanax* SF and CF are able to successfully breed together and produce viable offspring (reviewed in [14]), this observation makes *Astyanax mexicanus* an ideal system with which to study the evolutionary and genetic basis of scleral ossification in teleosts.

Previous research into the genetic basis of scleral ossification in *Astyanax* has found that the loss of scleral ossification in members of the Pachón cave population is inherited recessively through the epistatic interaction of three genes [13]. O'Quin *et al*. [13] crossed CF from Pachón cave with SF from Balmorhea State Park, Texas (Tx), and found a highly skewed distribution of F2 hybrids possessing scleral ossicles. Such a skewed distribution is characteristic of an epistatic threshold model of inheritance in which individuals must inherit recessive alleles at multiple genes before scleral ossification is lost. O'Quin *et al*. [13] also used quantitative trait locus (QTL) mapping to identify the location of these genes; however, due to the complex epistatic nature of this trait, as well as the relatively small number of individuals used (*n* = 196), they could only detect one QTL for overall ossicle size, and none for scleral ossicle number (S1 File).

The present study focuses on expanding the sample size of the previous analysis by O’Quin *et al*. [13] using an additional 225 F2 hybrids originally analyzed for craniofacial bone morphology in Gross *et al*. [15]. These additional hybrids are derived from a cross of CF from Pachón (Pa) cave with SF from Río Valles in Mexico (Mx) [16]. Although the SF populations used in these two studies are members of distinct epigean lineages that independently invaded Central America approximately 1–5.9 million years apart [17], the use of CF from the same population (Pachón) implies that the same derived alleles for scleral ossification should segregate in both crosses. We analyzed these CF(Pa) x SF(Mx) hybrids from Gross *et al*. [15] individually and in combination with the CF(Pa) x SF(Tx) hybrids from O'Quin *et al*. [13] in order to (1) confirm the recessive threshold model of inheritance, (2) refine the number of genes involved in scleral ossicle formation, and (3) increase the power to detect QTL responsible for scleral ossicle formation. The results support a epistatic threshold model of inheritance, though only two genes are implicated in the CF(Pa) x SF(Mx) cross from Gross *et al*. [15]. The QTL analysis of the combined datasets identified two genomic locations strongly associated with scleral ossicle number on linkage groups 4.2 and 21, and a weaker QTL on linkage group 4.1. This is the second study to estimate the number of genes responsible for scleral ossification in *Astyanax*, and the first to successfully map their genomic location. These results will aid in the understanding of how scleral ossicles have evolved in this and other teleost fish species.

## Materials and methods

### Ethics statement

The fish used in this study were bred and collected as part of several previously published studies (e.g., [13, 15,16,18]). In each case, the animals were bred and collected with the approval of the Institutional Animal Care and Use Committee of either the University of Maryland, College Park (approval for CF(Pa) x SF(Tx) F2 hybrids via William R. Jeffery; see O'Quin *et al*. [13]) or New York University (approval for CF(Pa) x SF(Mx) F2 hybrids via Richard Borowsky; see Protas *et al*. [16]). Prior to sampling and staining (see below), all fish were euthanized using a lethal dose of 250 mg/L buffered MS-222.

### Sampling

The sample size for this study included two different groups of surface fish (SF) and cavefish (CF) hybrids. The first group consisted of 225 individuals of >6 month old adult *Astyanax mexicanus* CF(Pa) x SF(Mx) F2 hybrids that were originally generated in Protas *et al*. [16] and analyzed for craniofacial bone in Gross *et al*. [15]. The SF used in Protas *et al*. [16] were obtained from Mexican populations near Río Valles (Mx), while the CF were derived from Pachón cave (Pa). We performed all analyses using the above dataset individually and then in combination with a second group of CF(Pa) x SF(Tx) hybrids. The second group consisted of 196 *Astyanax mexicanus* CF(Pa) x SF(Tx) F2 hybrids that were originally generated by Yoshizawa *et al*. [18] and stained for scleral ossicles in O’Quin *et al*. [13]. The fish from this sample were adults between 2–5 years old. The CF used in Yoshizawa *et al*. [18] were also derived from Pachón cave; however, the SF were obtained from a Texas population in Balmorhea State Park (Tx). Because the two SF populations are members of distinct epigean lineages (reviewed in [17]), it is possible that these groups differ in the number of alleles affecting sclera ossification. Since both crosses use individuals from the same Pachón cave population, most, if not all, of the alleles responsible for the loss of scleral ossicles should be shared in both crosses.

### Staining

Both hybrid groups used in this study were stained to visualize ossified bone. The 225 CF(Pa) x SF(Mx) F2 hybrids from Gross *et al*. [15] were cleared and stained using Alizarin red, stored in sterile glycerol at 4°C, and then visualized with a Leica M205FA stereomicroscope at 7.81x magnification with a DFC310FX camera. The 196 CF(Pa) x SF(Tx) F2 hybrids from O’Quin *et al*. [13] were stained with Alizarin red and Alcian blue following standard protocols [19]. These samples were temporarily stored in a solution of 1x PBS and 0.5% sodium azide at 4°C until imaged one week later. Additional information on the staining and storage of both sets of samples is available in Gross *et al*. [15] and O’Quin *et al*. [13].

### Quantifying scleral ossification

**Fig 1:**
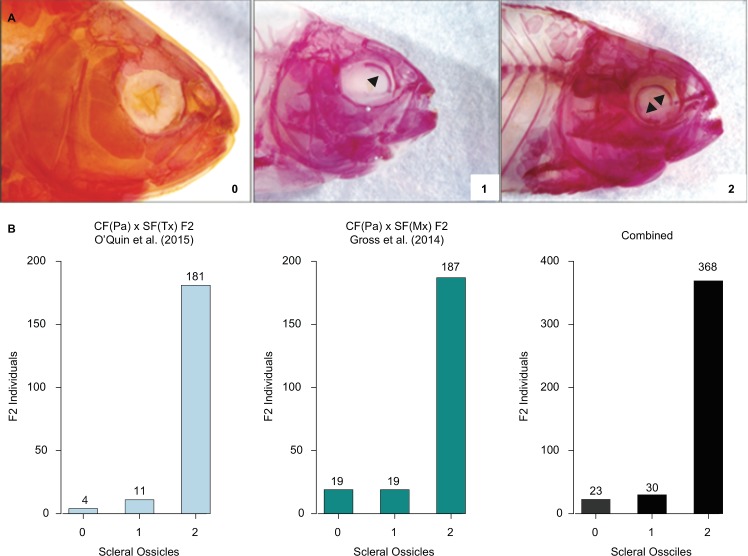
Skewed distribution of scleral ossicles in CF x SF F2 hybrids. (**A**) Example CF(Pa) x SF(Mx) F2 progeny from Gross *et al*. [15] with 0, 1, and 2 scleral ossicles. (**B**) Distribution of scleral ossicles among CF(Pa) x SF(Tx) F2 originally published in O'Quin *et al*. [13], from CF(Pa) x SF(Mx) F2 from Gross *et al*. [15], and the combined F2 from both analyses. In each case, the skewed distributions of F2 progeny with two scleral ossicles is consistent with hypothesis that multiple genes control scleral ossicle formation through an epistatic threshold model of inheritance.

To determine the number of scleral ossicles present in each individual, we examined the anterior and posterior regions of the eye for ossification. For the CF(Pa) x SF(Mx) F2 hybrids from Gross *et al*. [15], we examined only the right eye because cavefish can exhibit asymmetry in craniofacial morphology [15], and because the left eye was removed from most individuals prior to analysis. Similarly, we examined only the left eye of the CF(Pa) x SF(Tx) F2 hybrids from O’Quin *et al*. [13] since the right had was previously used to assess retinal degeneration [13]. Although different eyes were inspected for each group, it should not affect the final results since we do not expect one side to be consistently different from the other. Each eye was scored zero (0) for no scleral ossification, one (1) for partial ossification from one scleral ossicle (always the anterior ossicle), or two (2) for ossification from both anterior and posterior scleral ossicles, which may or may not have fused into a single ring (Fig 1). These qualitative values were recorded and then depicted as a bar graph to view the distribution of scleral ossicles in order to confirm the threshold model of inheritance.

### Estimating the number of genes

Scleral ossicle loss is controlled by a genetic threshold of approximately three genes [13]. In a genetic threshold, multiple loci must be recessive in order to produce the mutant phenotype. We analyzed the proportion of F2 progeny from Gross *et al*. [15] with (1+) and without (0) scleral ossicles against a model of inheritance assuming a genetic threshold at one to four genes. Using Microsoft Excel®, a χ^2^ test was performed on the dataset containing the 225 CF(Pa) x SF(Mx) F2 hybrids and then repeated using these 225 individuals in combination with the 196 CF(Pa) x SF(Tx) F2 hybrids from O’Quin *et al*. [13]. Hypotheses for one to four genes were rejected if the p-value fell below 0.05.

### Creating a composite linkage map

To identify the genomic location of the genes associated with the number of scleral ossicles, we generated a composite genetic linkage map of the *Astyanax mexicanus* genome using microsatellite markers genotyped in both the CF(Pa) x SF(Mx) F2 hybrids from Gross *et al*. [15] and the CF(Pa) x SF(Tx) F2 hybrids from O'Quin *et al*. [13]. In order to keep the naming of linkage groups consistent between the analysis of Gross *et al*. (2014) and O’Quin *et al*. [13], we identified orthologous groups between the genetic linkage maps of both studies, and renamed the linkage groups in Gross *et al*. [15] to match the naming conventions used in O’Quin *et al*. [13] and O’Quin *et al*. [20], since the latter studies used a more complete linkage map. To identify orthologous linkage groups, we searched for microsatellite markers shared in both studies to identify homologous markers, and then matched the names accordingly. For example, linkage group (LG) 5 from Gross *et al*. [15] became LG 1 from O'Quin *et al*. [13] because they shared markers Am210a, Am205e, OCA2, etc. There were a few instances where multiple linkage groups from Gross *et al*. [15] were either combined or given similar names to match a corresponding single linkage group from O’Quin *et al*. [13], e.g. LG 4.1 and 4.2 (S1 Table). In a few cases, no homologous markers were shared between the two studies, such as with LGs 6, 8, and 29 from Gross *et al*. [15]. These LGs were not included in the final analysis. Note that we did not change the order or distance of individual markers on each LG as reported in Gross *et al*. [15]. The final map for the combined linkage map included 105 genetic markers distributed across 27 LGs totaling 1040.83 cM. A complete list of the LGs and their identities is included in S1 Table.

### Identifying gene location through QTL mapping

We performed three QTL analyses for scleral ossicle number using the combined linkage map in the program R/qtl [21]. For the first analysis, we re-analyzed the 196 CF(Pa) x SF(Tx) F2 hybrids from O'Quin *et al*. [13] using the reduced set of 105 microsatellite markers from the combined map. This re-analysis was performed so that the results of O'Quin *et al*. [13] could be readily compared to those from the 225 CF(Pa) x SF(Mx) F2 hybrids from Gross *et al*. [15]. We do not expect the results of this re-analysis to change relative to those originally published in O'Quin *et al*. [13], although the dramatically reduced number of genetic markers (105 vs. 698) could provide additional statistical power to detect weak QTL, since fewer statistical comparisons must be corrected for. For our second analysis, we analyzed the 225 CF(Pa) x SF(Mx) F2 hybrids from Gross *et al*. [15] and, finally, for our third analysis we performed a combined analysis of all 421 CF(Pa) x SF F2 progeny from both studies. For all analyses, we treated ossicle number as an ordinal trait with three states: 0, 1, and 2.

In each QTL analysis, we performed an initial scan for QTL assuming the presence of a single QTL per linkage group without interactions using the function *scanone* and Haley-Knott regression. This analysis calculates the logarithm of the odds (LOD) of association between the average number of scleral ossicles and the genotypes at each genetic marker. We evaluated the statistical significance of the LOD scores by calculating the 95^th^ percentile of genome-wide maximum penalized LOD scores for each phenotype using 1,000 random permutations of the genotypic and phenotypic data. Because scleral ossification is a complex trait controlled by epistatic interactions among multiple loci [13], this initial scan is not expected to be powerful enough to detect all loci, although it can be useful for giving a genome-wide view of potential QTL. We then performed a second scan for multiple, potentially interacting QTL using the functions *scantwo* and *stepwiseqtl* following the same criteria as above. This second analysis is much more powerful, especially for complex traits like scleral ossification, and gives a detailed, LG-level view of individual and interacting QTL. More information on our QTL mapping procedure is available in O’Quin *et al*. [13]. We did not use standard length or age as covariates during the QTL analysis because overall ossicle number is presumably determined early in development and is not influenced by the size or age of the adult animal [12]. In addition, while the length of each scleral ossicle may be affected by age [8,13], we did not examine ossicle length in this study. The genotypic and phenotypic data used to identify QTL from the CF(Pa) x SF(Mx) F2 hybrids from Gross *et al*. [15] samples and the combined CF x SF F2 hybrids from both O'Quin *et al*. [13] and Gross *et al*. [15] are available in S2 and S3 Tables, respectively.

## Results

### Frequency of scleral ossification

We obtained photographs of 225 Pachón cavefish (CF(Pa)) x Mexican surface fish (SF(Mx)) F2 hybrids stained with alizarin red from Gross *et al*. [15]. We analyzed these samples visually for the presence of scleral ossicles. Each individual received a score of zero (0), one (1), or two (2) based on the number of scleral ossicles in the right eye. Our previous study [13] found that the vast majority of CF(Pa) x Texas surface fish (SF(Tx)) F2 hybrids possess two scleral ossicles, while relatively few possess only one or even no ossicles [13] (Fig 1). Consistent with these previous results, we found that most CF(Pa) x SF(Mx) F2 hybrids also possess two ossified scleral ossicles like SF (n = 187), while fewer possess only one scleral ossicle (n = 19) or, like CF, no scleral ossicles at all (n = 19; Fig 1). Combining the hybrids from these two studies together strengthened this trend. Of the 421 F2 hybrids sampled overall, the majority exhibited two scleral ossicles (n = 369), while relatively few hybrids exhibited either one (n = 30) or no ossicles (n = 23; Fig 1B). The distribution of roughly 16:1:1 for 2, 1, and 0 scleral ossicles respectively indicates that multiple genes are involved in scleral ossicle formation and is consistent with an epistatic threshold model of inheritance.

### Estimating the number of genes

Our previous analysis determined that the frequency of CF(Pa) x SF(Tx) F2 hybrids with (1+) and without (0) scleral ossicles was consistent with a model of epistatic threshold inheritance caused by three genes [13]. We performed a χ^2^ test on the CF(Pa) x SF(Mx) F2 hybrids from Gross *et al*. [15] and again in combination with the CF(Pa) x SF(Tx) F2 hybrids from O’Quin *et al*. [13] to confirm this model. We compared the observed ratio of F2 offspring with (1+) and without (0) scleral ossicles to those expected for threshold inheritance at one, two, three, and four genes. For the 225 CF(Pa) x SF(Mx) F2 hybrids obtained from Gross *et al*. [15], the observed frequency of F2 with and without scleral ossicles was 206:19 or 10.84:1 (Fig 1). These results were consistent with a two-locus model of threshold inheritance (Expected frequency: 15:1, χ^2^ = 1.85, df = 1, *P* = 0.168), but not a one, three, or four gene model (all *P* < 0.001; Table 1). Similarly, for the results of the combined dataset of all 421 hybrids, the observed frequency of F2 with and without scleral ossicles was 399:23 or 17.35:1, which was also consistent with a two-locus model of threshold inheritance (Expected frequency: 15:1, χ^2^ = 0.46, df = 1, *P* = 0.544; Table 2). The observed proportions of F2 with 0, 1, and 2 scleral ossicles differ significantly between the CF(Pa) x SF(Tx) F2 hybrids of O'Quin *et al*. [13] and the CF(Pa) x SF(Mx) F2 hybrids of Gross *et al*. [15] (χ^2^ = 50.53, df = 2, *P* < 0.0001; Fig 1). Instead, the results show that the loss of scleral ossicles follows a two-gene model of threshold inheritance in the CF(Pa) x SF(Mx) cross, in contrast to the three-gene model observed in the CF(Pa) x SF(Tx) cross [13].

**Table 1:** Chi-square (χ^2^) analysis of observed and expected ratios of CF(Pa) x SF(Mx) F2 progeny from Gross *et al*. [15] with and without scleral ossicles due to a genotypic threshold at 1–4 loci.

Loci	Frequency	Ossicles Present	Ossicles Absent	χ^2^	P-value
Observed	10.84:1	206	19	NA	NA
1	3:1	169	56	32.89	< 0.001
2	15:1	211	14	1.85	0.168
3	63:1	221	4	69.28	< 0.001
4	255:1	224	1	375.08	< 0.001

**Table 2:** Chi-square (χ^2^) analysis of observed and expected ratios of the combined CF(Pa) x SF(Mx) F2 progeny from Gross *et al*. [15] and the CF(Pa) x SF(Tx) F2 progeny from O’Quin *et al*. [13] with and without scleral ossicles due to a genotypic threshold at 1–4 loci.

Loci	Frequency	Ossicles Present	Ossicles Absent	χ^2^	P-value
Observed	17.35:1	399	23	NA	NA
1	3:1	317	106	86.02	< 0.001
2	15:1	396	26	0.46	0.544
3	63:1	415	7	41.47	< 0.001
4	255:1	420	2	277.6	< 0.001

### Identifying the location of genes

**Fig 2:**
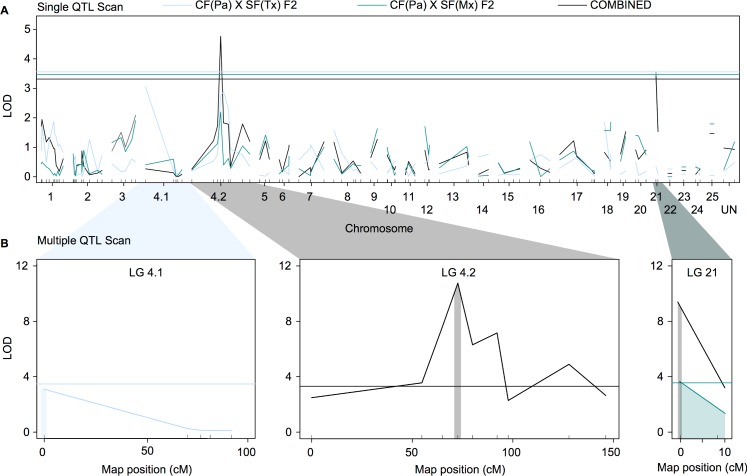
Three quantitative trait loci (QTL) for scleral ossicle number on *Astyanax* linkage groups 4.1, 4.2, and 21. (**A**) A scan for a single QTL for scleral ossicle number per chromosome in the CF(Pa) x SF(Tx) F2 dataset from O'Quin *et al*. [13], the individual CF(Pa) x SF(Mx) F2 dataset from Gross *et al*. [15], and the combined datasets. This scans potential QTL on LG 4.2 and 21. (**B**) A more robust scan for multiple QTL across all three analyses confirmed the presence of QTL on LG 4.1 in the CF(Pa) x SF(Tx) F2 from O'Quin *et al*. [13], as well as on LG 4.2 and LG 21 in the CF(Pa) x SF(Mx) F2 from Gross *et al*. [15] and the combined datasets. Shaded area represents the 95% confidence interval for the location of the QTL. An additional interaction was detected between the QTL on LG 4.2 and 21 (Table 3).

**Fig 3:**
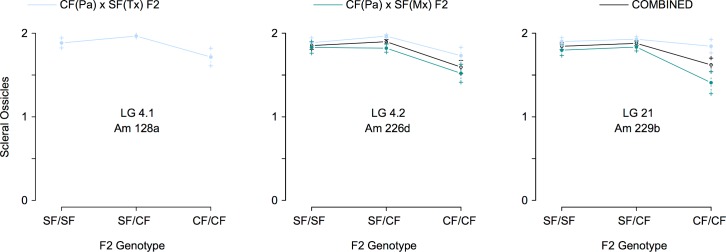
Recessive inheritance of scleral ossicles at all QTL positions. Effect plots displaying average scleral ossicle number for each genotypic class observed in the CF x SF F2 hybrids from all three analyses. In each case, F2 hybrids with two cavefish alleles have fewer scleral ossicles on average than those with either one or two surface fish alleles. This pattern is consistent with the recessive inheritance of scleral ossicle loss in CF [13].

To identify the location of genes and mutations associated with scleral ossicle number, we used genotypes and a composite genetic linkage map for the CF(Pa) x SF(Tx) F2 hybrids from O'Quin *et al*. [13] and the CF(Pa) x SF(Mx) F2 hybrids in Gross *et al*. [15] to search for quantitative trait loci (QTL) associated with scleral ossicle number. We scanned both datasets, both individually and in combination, using an initial single-QTL scan and a second multiple-QTL scan in R/qtl [21]. The initial scan of O’Quin *et al*. [13] assumed the presence of a single QTL per linkage group (LG) and revealed two weak peaks on LGs 4.1 and 4.2, but these did not surpass the threshold logarithm of the odds (LOD) score to be significant (Fig 2A). These peaks increased in significance following the *stepwise* scan for multiple QTL, resulting in the identification of a single significant QTL on LG 4.1 (Fig 2B). This QTL is located at the distal end of LG 4.1 at 0.00 cM, corresponding the microsatellite marker Am128a (LOD = 3.04, *P* = 0.001). This single QTL accounts for 6.86% of the variation in scleral ossicle number among the CF(Pa) x SF(Tx) F2 hybrids from O'Quin *et al*. [13] (Table 3). Examination of the effect plot for the peak marker on LG 4.1 reveals a recessive pattern of inheritance, whereby CF(Pa) x SF(Tx) F2 hybrids with two CF alleles possess fewer scleral ossicles on average than F2 with one or more SF alleles (Fig 3). This effect is consistent with the expected recessive inheritance of scleral ossicle loss (O'Quin *et al*. 2015). Although this same region exhibited elevated LOD in the original scan of QTL for scleral ossicle number published in O'Quin *et al*. [13], it did not rise above the genome-wide threshold of significance, probably due to the greater number of microsatellite comparisons performed in that study (698 vs. 105).

**Table 3:** Summary statistics of all quantitative trait loci (QTL) for scleral ossification detected in this study. LOD = logarithm of the odds. PVE = percent variance explained.

Cross	LG	Marker	LOD	PVE	P-value	Additive Effect	Dominance Effect
CF(Pa) x SF(Tx) F2 (O'Quin *et al*. [13])	4.1	Am128a	3.04	6.87%	0.0010	0.085	0.167
CF(Pa) x SF(Mx) F2 (Gross *et al*. [15])	21	Am229b	3.74	7.38%	0.0002	0.196	0.237
Combined	4.2	Am226d	10.8	10.7%	8.5x10^-9^	0.136	0.164
	21	Am229b	9.28	7.38%	1.3x10^-7^	0.084	0.139
	4.2: 21	Am226d: Am229b	6.21	6.01%	1.2x10^-5^	-0.172: -0.072	-0.264: -0.219

The initial scan of the CF(Pa) x SF(Mx) F2 hybrids from Gross *et al*. [15] similarly revealed a weak QTL peak on LG 21 (LOD = 3.74, *P* = 0.039; Fig 2A). The more robust multi-locus *stepwise* QTL analysis strengthened this result, but did not identify any additional QTL (Fig 2B). This QTL is centered on the distal end of LG 21 from 0.00–6.15 cM, corresponding to microsatellite marker Am229b (LOD = 3.75, *P* = 0.0002) (Table 3). Overall, this QTL explained 7.38% of the variance in scleral ossicle number among the CF(Pa) x SF(Mx) F2 hybrids from Gross *et al*. [15], and its effect plot also supported recessive inheritance (Fig 3). Interestingly, the same marker exhibits a slight pattern of recessive inheritance in the CF(Pa) x SF(Tx) F2 hybrids from O'Quin *et al*. [13], though the effect is much stronger among the CF(Pa) x SF(Mx) F2 hybrids from Gross *et al*. [15] (Fig 3).

The combined analysis of all 196 CF(Pa) x SF(Tx) F2 hybrids from O'Quin *et al*. [13] and 225 CF(Pa) x SF(Mx) F2 hybrids from Gross *et al*. [15] greatly increased the power to detect interacting QTL for scleral ossification. An initial scan of the 421 F2 hybrids from both studies resulted in two significant QTL on LGs 4.2 (LOD = 4.76, *P* < 0.001) and 21 (LOD = 3.43, *P* = 0.037; Fig 2A). These peaks increased dramatically in significance following the multi-locus *stepwise* analysis (Fig 2B), indicating the combined strength of both datasets. The peak on LG 4.2 is located at 72.6 cM, corresponding to microsatellite marker Am226d (LOD = 10.76, *P* = 8.5 x 10^−9^; Table 3). This QTL accounted for 10.67% of the variance in scleral ossicle number among all CF(Pa) x SF F2 hybrids, and the effect plot indicated that F2 hybrids with two CF alleles had fewer scleral ossicles on average than those with either one or two SF alleles (Fig 3). This result matches the expected trend that the loss of scleral ossicles is inherited in a recessive manner [13]. Importantly, this same region exhibits elevated LOD in both the CF(Pa) x SF(Tx) F2 hybrids from O'Quin *et al*. [13] and the CF(Pa) x SF(Mx) F2 hybrids from Gross *et al*. [15] (Fig 2), but the effect was not large enough to detect statistically in either cross individually (Fig 3). The second significant peak on LG 21 corresponds to the same QTL identified in the individual analysis of the CF(Pa) x SF(Mx) F2 from Gross *et al*. [15] (Fig 2B). As in that cross, this QTL is located at the distal end of LG 21 and corresponds to marker Am229b (LOD = 9.43, *P* < 1.3 x 10^−7^; Table 3). This QTL accounts for 9.28% of the variance in scleral ossicle number, and the effect plot also matches the expected recessive trend that hybrids with two CF alleles have a reduced number of scleral ossicles (Fig 3). Finally, a third QTL was accounted for by the interaction of the QTL on LG 4.1 and 21 (LOD 6.21, *P* = 1.22 x 10^−5^; Table 3). This interaction indicates that the phenotypes observed at the first genetic marker depend on the genotypes at the second genetic marker, which is characteristic of epistatic gene interactions. This epistatic interaction accounts for an additional 6.21% of the variance in scleral ossicle number among all the CF(Pa) x SF F2 hybrids (Table 3). Together, the three QTL identified in the combined analysis of the CF(Pa) x SF F2 hybrids from O'Quin *et al*. [13] and Gross *et al*. [15] account for 26.40% of the variance in scleral ossicle number among these hybrids.

## Discussion

### Scleral ossicle number exhibits epistatic threshold inheritance

We analyzed the CF(Pa) x SF(Mx) F2 hybrids from Gross *et al*. [15] for scleral ossification and found a highly skewed ratio of approximately 10:1:1 for two (2), one (1), and zero (0) scleral ossicles, respectively. Previously, O’Quin *et al*. [13] found a ratio of approximately 45:3:1 for CF(Pa) x SF(Tx) F2 hybrids. While these ratios are statistically different (χ^2^ = 50.53, df = 2, *P* < 0.0001), they are similar in that they are both highly skewed towards F2 hybrids with two scleral ossicles (see also Fig 1B). The slight difference between the results of Gross *et al*. [15] and our previous results in O’Quin *et al*. [13] is likely due to different numbers of genes affecting scleral ossification segregating in the two crosses (for more details, see below). Finally, we combined the F2 progeny from both Gross *et al*. [15] and O’Quin *et al*. [13] and found a ratio of approximately 16:1:1 for the combined dataset. Overall, the distribution of scleral ossicles among the F2 of both crosses, individually as well as in combination, is consistent with an epistatic threshold model of inheritance, whereby individuals must inherit recessive alleles at multiple loci before they express a mutant phenotype. For the CF(Pa) x SF(Tx) F2 hybrids sampled in O'Quin *et al*. [13], in which three genes contribute to scleral ossification, the pattern is best described as multiple or triple dominant epistasis, since a dominant wild-type allele at any one of three loci is sufficient to produce scleral ossicles, while individuals that inherit two recessive alleles at all three loci will lose scleral ossicles. For the CF(Pa) x SF(Mx) F2 hybrids sampled in Gross *et al*. [15], in which two genes contribute to scleral ossification (Table 1), this inheritance pattern is best described as duplicate dominant epistasis. We examined other models of epistasis for two genes using the CF(Pa) x SF(Mx) F2 hybrids from Gross *et al*. [15], but the observed results were only consistent with the expectations for duplicate dominant epistasis (S4 Table). The observation that the inheritance of scleral ossicles is consistent with a simple presence/absence model of duplicate dominant epistasis may suggest that the presence of one versus two scleral ossicles is not due to any genetic difference, but rather some environmental or developmental variation. Previous research has shown that the dorsal scleral ossicle forms prior to the ventral ossicle in *Astyanax mexicanus* [8]. Therefore, F2 hybrids with only a single dorsal ossicle may simply not have developed their second ventral ossicle yet, although it is not yet possible to reject more complex genetic and environmental factors. Threshold inheritance has also been described for other traits in *Astyanax*, including eye size, melanophore number, taste bud number, and feeding behavior [22].

### Two—Three loci control the loss of scleral ossicles

By comparing the observed ratio of F2 progeny with (1+) and without (0) scleral ossicles to those expected for threshold inheritance at one to four loci, we were able to estimate the number of genes likely involved in the loss of scleral ossicles. Our analysis of the CF(Pa) x SF(Mx) F2 hybrids sampled in Gross *et al*. [15] showed that the loss of scleral ossification is consistent only with a two-locus model of inheritance (Table 1). This contradicts the results for CF(Pa) x SF(Tx) F2 hybrids obtained by O’Quin *et al*. [13], which identified a three-locus model of inheritance for scleral ossicles. This difference could be a result of the different surface fish populations used in each analysis. O’Quin *et al*. [13] crossed Pachón cavefish (CF) with surface fish (SF) from Texas, while Gross *et al*. [15] crossed Pachón CF with SF from Mexico. Texas SF descended from *Astyanax mexicanus* populations that invaded Central America approximately 2–5 Mya. This ‘old’ lineage of SF gave rise to several CF populations, including Pachón, before going extinct in most regions except Texas [17]. In contrast, the current Mexican SF lineages evolved from a separate invasion that occurred around 2.1 Mya [17]. The ‘young’ SF gave rise to additional CF populations located to the north and west of the Pachón locality [17]. Importantly, however, regardless of which SF population is used, both O'Quin *et al*. [13] and Gross *et al*. [15] used CF from the same population, Pachón, implying that the same derived alleles affecting scleral ossification should segregate in both crosses.

It is possible that, since the SF used in each cross are derived from distinct lineages, that different numbers of genetic factors controlling scleral ossification are fixed in each group. For example, the Mexican SF from Río Valles could conceivably be fixed for recessive CF alleles at one of the responsible loci, for example, the weak QTL on LG 4.2 detected only in the CF(Pa) x SF(Tx) F2 hybrids from O'Quin *et al*. [13]. Since the presence of scleral ossicles is controlled by multiple dominant epistasis, these recessive alleles would not impact the normal formation of scleral ossicles in this group. Alternatively, it is possible that members of the Pachón CF population may themselves vary in the number of mutations in these genes; however, we think this explanation is less likely since all members of Pachón CF so far surveyed lack scleral ossicles, which would suggest that they are fixed for recessive mutations at all responsible loci. In either case, that different numbers of loci segregate between the different populations is not in itself surprising, since surface fish are genetically diverse [17,23], and different cavefish populations also exhibit dramatic variation in the number and identity of genes contributing to eye and melanin degeneration [22]. Whether similar numbers of genes and genetic interactions are responsible for the loss of scleral ossification in other *Astyanax* cave populations, such as those from Curva, Los Sabinos, and Molino caves, remains to be seen since the inheritance of scleral ossicles has not been studied in these groups [13]. Because cave phenotypes have evolved independently in many of these linages and exhibit considerable variation in the number and type of genes mutated (reviewed in [14]), it would not be surprising to observe diversity in the genetic factors controlling scleral ossification as well.

This is only the second study to estimate the number of genes responsible for the loss of scleral ossicles among vertebrates [13]. Although the development and evolution of scleral ossicles have been described in several other organisms, including turtles, lizards, and birds [1], their inheritance has not been studied systematically. The only comparable example in other vertebrates is a series of studies on the *scaleless* mutant in chickens [24]. *Scaleless* chicks exhibit abnormal keratinization, resulting in an almost complete lack of downfeathers, scales, spurs, and scleral ossicles [24–26]. Using a series of crosses with heterozygous *scaleless* chickens, Abbott and Asmundson [24] determined that the *scaleless* phenotype was caused by recessive alleles at a single autosomal gene (one-locus threshold model of inheritance). The difference in the number of genes involved in *Astyanax* versus *scaleless* chickens may be due to the difference in the types of ossification involved. In chickens, scleral ossification stems directly from a membranous bone [27], while in teleosts scleral ossicles form indirectly via cartilage replacement [8]. Interestingly, Wells *et al*. [5] recently used genome-wide association mapping to isolate the genetic basis of *scaleless* to a single nonsense mutation in the gene *fgf20*. The results of that study indicate that in birds, at least, scleral ossicle formation is either the direct or indirect result of *fibroblast growth factor* signaling. Other studies of scleral ossicle induction in chicks have identified an important role for *hedgehog* and *bone morphogenic protein* signaling as well [4].

### Genes responsible for scleral ossification are located on *A*. *mexicanus* LGs 4 and 21

Quantitative trait loci (QTL) analysis of both datasets, both individually and in combination, allowed us to identify the genomic location of the three genes associated with scleral ossicle number (Fig 3). For the individual CF(Pa) x SF(Tx) F2 hybrids from O’Quin *et al*. [13], we found evidence of a weak QTL on *Astyanax* linkage group (LG) 4.1. This same region exhibited elevated LOD in our previous analysis [13], but it was not statistically significant at the genome-wide threshold level, probably as a result of the large number of comparisons made in that study. For the CF(Pa) x SF(Mx) F2 hybrids from Gross *et al*. [15], we found one QTL on LG 21. The combined analysis of both datasets resulted in a large increase in power to detect individual as well as interacting QTL, including an additional QTL on LG 4.2. This genomic region also exhibited elevated LOD in both the CF(Pa) x SF(Tx) cross of O'Quin *et al*. [13] and the CF(Pa) x SF(Mx) cross of Gross *et al*. [15], but was not significant in either cross individually. The dramatic increase in LOD observed for the two QTL on LG 4.2 and 21, as well as the interaction between them, supports the contention that the same alleles affecting scleral ossification are segregating in both crosses due to their use of the same CF population from Pachón. Notably, the effect plots for genetic markers at each QTL all support a recessive threshold model of inheritance, whereby hybrids must inherit two CF alleles at each marker before exhibiting a reduction in the number of scleral ossicles, and the presence of a significant interaction QTL in the combined dataset is also consistent with this type of epistatic interaction. These results support our previous and current results that scleral ossicle loss is inherited in a recessive manner through epistatic interactions among 2–3 genes in Pachón cavefish, depending on which surface fish lineage they are compared to [13] (Table 1).

Interestingly, genetic markers located within all three QTL regions have been associated with phenotypic differences in other *Astyanax* crosses as well, though, notably, none for eye degeneration. For example, both genetic markers Am128a and Am226d on LGs 4.1 and 4.2, respectively, are associated with a QTL for relative condition [27]. Although the peak QTL marker on LG 4.1, Am128a, is adjacent to a QTL for lens size [27] and around 7 cM from the gene *fgf8* [28], this QTL for scleral ossicle number appears to be independent of any existing QTL for eye size or bone development. Somewhat similarly, the peak genetic marker for the QTL on LG 21, Am229b, is located at the absolute distal end of the confidence interval for a QTL associated with the combined effects of eye size, melanophore number, number of anal fin rays, peduncle depth, and width of suborbital bone SO_3_ [27]. The close association of QTL for these traits is consistent with a potentially pleiotropic basis for eye and scleral ossicle development [18,29]. However, the lack of direct overlap between any genetic markers for scleral ossification and those previously associated with eye or lens development is notable since previous research has shown that the presence or absence of a lens during eye development can influence the extent of scleral ossification in *Astyanax* CF and SF [11–12]. The observed lack of direct overlap between scleral and eye QTL suggests that the genetic mechanisms responsible for the loss of scleral ossification in Pachón cavefish are partially or even wholly distinct from those contributing to eye regression. This conclusion is consistent with previous observations of independence between eye development and other craniofacial bones [11], as well as the presence of cavefish lineages that maintain scleral ossification in the presence of significant levels of eye degeneration [12–13]. This observation may also explain why many teleost species lack scleral ossicles while retaining large, functional eyes [6,9].

The small number of genetic markers used in our combined analysis makes it difficult to infer candidate genes for scleral ossification. Two possible candidates are the genes *fgf8a* and *pax2a*, which are both located within 10 cM of microsatellite marker Am128a on LG 4.1 [28]. As mentioned earlier, previous studies have linked mutations in *fgf20* to the loss of scleral ossicles in *scaleless* chicks [5]. Similarly, previous studies have also shown that *fgf8* and *pax2* are altered in cavefish development, likely as a result of the expanded *shh* signaling that results in lens degeneration [30–32]. *Fgf8* and *shh* have also been shown function together in the development of other eye and craniofacial features in vertebrates, including beak shape in chickens and jaw shape in zebrafish [3,33]. Thus, it is possible that these two genes act synergistically to direct the ossification of scleral ossicles in *Astyanax*. Such a result could suggest that the scleral ossicles of teleosts, birds, and reptiles share a common molecular basis despite their anatomical and developmental differences. Future work will determine what role, if any, these and other candidate genes play in teleost scleral ossicle development.

## Conclusions

This is the second study to identify the number of genes responsible for scleral ossification in teleost fishes, and the first to successfully map their genomic location. We have determined that the loss of scleral ossicles follows a two—three locus epistatic threshold model of inheritance in the teleost *Astyanax mexicanus*. It is possible that two genes are involved in the Gross *et al*. [15] cross while three are involved in the O’Quin *et al*. [13] cross because these crosses used surface fish with distinct evolutionary histories [17]. By combining these crosses, we enhanced our ability to detect QTL for this trait and found two regions located on *Astyanax* linkage groups 4.2 and 21, as well as a weaker QTL on linkage group 4.1. Future work will aim to fine map these QTL by performing a backcross between CF x SF F1 and cavefish while using hundreds of additional genetic markers. Scleral ossification is a complex trait with limited characterization of its genetic basis. This study provides insight into the number and location of genes controlling the formation of scleral ossicles in *Astyanax mexicanus*, which can be applied to other teleost fishes as well.

## Supporting information

S1 Table Composite CF(Pa) x SF linkage map. Comparison of homologous linkage groups from CF(Pa) x SF(Mx) F2 hybrid and CF(Pa) x SF(Tx) F2 hybrid linkage maps published in Gross *et al*. [15] and O’Quin *et al*. [13].
(DOCX)

S2 Table Phenotypic and genotypic data used to detect QTL for scleral ossification in the CF(Pa) x SF(Mx) F2 from Gross *et al*. [15] including genetic distances. (CSV)

S3 Table Phenotypic and genotypic data used to detect QTL for scleral ossification in the combined CF(Pa) x SF(Mx) F2 from Gross *et al*. [15] and CF(Pa) x SF(Tx) F2 from O'Quin *et al*. [13] including genetic distances. (CSV)

S4 Table Chi-square (χ^2^) analysis of observed and expected ratios of the CF(Pa) x SF(Mx) F2 progeny from Gross *et al*. [15]. Expected ratios for 0, 1, and 2 scleral ossicles estimated interactions of two genes caused by epistasis of one (single) or two (duplicate) loci.
(DOCX)

S1 File Analysis of scleral ossification among CF(Pa) x SF(Tx) F2 hybrids previously published in O'Quin et al. [13]. (PDF)
