# Supplementary material for: Two – three loci control scleral ossicle formation via epistasis in the cavefish Astyanax mexicanus
Source: PLoS One. 2017 Feb 9;12(2):e0171061. doi: 10.1371/journal.pone.0171061 (PMC5300192; doi:10.1371/journal.pone.0171061)
Supplement: S1 Table — Comparison of homologous linkage groups from CF(Pa) x SF(Mx) F2 hybrid and CF(Pa) x SF(Tx) F2 hybrid linkage maps published in Gross et al. [15] and O’Quin et al. [13]. (DOCX) [file pone.0171061.s001.docx]

**S1 Table.** **Composite CF(Pa) x SF linkage map.** Comparison of homologous linkage groups from CF(Pa) x SF(Mx) F2 hybrid and CF(Pa) x SF(Tx) F2 hybrid linkage maps published in Gross *et al*. [15] and O’Quin *et al*. [13].

| **Linkage Group (O’Quin *et al*. 2015)** | **Linkage Group (Gross *et al*. 2014)** | **Modification to Gross Linkage Map** | **Distance (cM) in Gross *et al*. (2014)** | **Distance (cM) in combined map.** |
| --- | --- | --- | --- | --- |
| 1 | 5 | NA | 33.8 to 88.8 | 55 |
| 2 | 7 | NA | 3 to 75.9 | 72.9 |
| 3 | 11 | NA | 25.7 to 85.3 | 59.6 |
| 4 | 13, 14 | 4.1 and 4.2 | 0.0 to 81.4 | 81.4 |
| 5 | 9 | NA | 20 to 44.2 | 24.2 |
| 6 | 10 | NA | 31.6 to 56.2 | 24.6 |
| 7 | 4 | NA | 24.8 to 88.1 | 63.3 |
| 8 | 21, 24 | 8 | 8.06 to 76 | 67.94 |
| 9 | 29, 27 | 9 | 29.6 to 46.1 | 16.5 |
| 10 | 20 | NA | 16.6 to 37.2 | 20.6 |
| 11 | 1 | NA | 36.8 to 61.1 | 24.3 |
| 12 | 18 | NA | 2.94 to 14.3 | 11.36 |
| 13 | 22, 28 | 13 | 0 to 74.1 | 74.1 |
| 14 | 26 | NA | 0 to 24.8 | 24.8 |
| 15 | 3 | NA | 1.58 to 55.4 | 53.82 |
| 16 | 15 | NA | 0 to 50.8 | 50.8 |
| 17 | 17, 2 | 17 | 8.11 to 96 | 87.89 |
| 18 | 25 | NA | 55.8 to 71.5 | 15.7 |
| 19 | 10, 1 | NA | 22 to 34.9 | 12.9 |
| 20 | 23 | NA | 2.27 to 28.5 | 26.23 |
| 21 | 27 | NA | 4.1 to 9.21 | 5.11 |
| 22 | NA | NA | 38.2 | 0 |
| 23 | 19 | NA | 64 | 0 |
| 24 | 16 | NA | 0 to 11.1 | 11.1 |
| 25 | 12 | NA | 56.3 | 0 |
| UN | 10, 29 | UN | 290 to 317 | 27 |
| NA | G6 | not included | NA | NA |
| NA | G8 | not included | NA | NA |
