# Supplementary material for: Two – three loci control scleral ossicle formation via epistasis in the cavefish Astyanax mexicanus
Source: PLoS One. 2017 Feb 9;12(2):e0171061. doi: 10.1371/journal.pone.0171061 (PMC5300192; doi:10.1371/journal.pone.0171061)
Supplement: S4 Table — Expected ratios for 0, 1, and 2 scleral ossicles estimated interactions of two genes caused by epistasis of one (single) or two (duplicate) loci. (DOCX) [file pone.0171061.s004.docx]

**S4 Table**. **Chi-square (χ^2^) analysis of observed and expected ratios of the CF(Pa) x SF(Mx) F2 progeny from Gross *et al*. [15]** .Expected ratios for 0, 1, and 2 scleral ossicles estimated interactions of two genes caused by epistasis of one (single) or two (duplicate) loci.

| **Epistasis** | **Frequency** | **2 Ossicles** | **1 Ossicles** | **0 Ossicles** | **χ^2^** | **P-value** |
| --- | --- | --- | --- | --- | --- | --- |
| Single Observed | 9.84 : 1 : 1 | 187 | 19 | 19 | NA | NA |
| Single Dominant | 12 : 3 : 1 | 169 | 42 | 14 | 16.45 | < 0.001 |
| Single Recessive | 9 : 3 : 4 | 127 | 42 | 56 | 66.27 | << 0.001 |
| Single Cumulative | 9 : 6 : 1 | 127 | 84 | 14 | 81.25 | << 0.001 |
| **Epistasis** | **Frequency** | **Ossicles Present** | **NA** | **Ossicles Absent** | **χ^2^** | **P-value** |
| Duplicate Observed | 10.84 : 1 | 206 | NA | 19 | NA | NA |
| Duplicate Dominant | 15 : 1 | 211 | NA | 14 | 1.85 | 0.397 |
| Duplicate Recessive | 9 : 7 | 127 | NA | 98 | 113.96 | << 0.001 |
