# Supplementary material for: Two – three loci control scleral ossicle formation via epistasis in the cavefish Astyanax mexicanus
Source: PLoS One. 2017 Feb 9;12(2):e0171061. doi: 10.1371/journal.pone.0171061 (PMC5300192; doi:10.1371/journal.pone.0171061)
Supplement: S1 File — (PDF) [file pone.0171061.s005.pdf]

PLOS | ONE

RESEARCH ARTICLE

Complex Evolutionary and Genetic Patterns Characterize the Loss of Scleral Ossification in the Blind Cavefish *Astyanax mexicanus*

Kelly E. O'Quin[1]*, Pooja Doshi[2], Anastasia Lyon[1], Emma Hoenemeyer[1], Masato Yoshizawa[3], William R. Jeffery[2]

1 Biology Program, Centre College, Danville, KY, 40422, United States of America, **2** Department of Biology, University of Maryland, College Park, MD, 20742, United States of America, **3** Department of Biology, University of Hawai'i at Manoa, Honolulu, HI, 96822, United States of America

* kelly.oquin@centre.edu

CrossMark
click for updates

OPEN ACCESS

Citation: O'Quin KE, Doshi P, Lyon A, Hoenemeyer E, Yoshizawa M, Jeffery WR (2015) Complex Evolutionary and Genetic Patterns Characterize the Loss of Scleral Ossification in the Blind Cavefish *Astyanax mexicanus*. PLoS ONE 10(12): e0142208. doi:10.1371/journal.pone.0142208

Editor: Harold A. Burgess, National Institutes of Health / NICHD, UNITED STATES

Received: August 10, 2015

Accepted: October 19, 2015

Published: December 9, 2015

Copyright: © 2015 O'Quin et al. This is an open access article distributed under the terms of the Creative Commons Attribution License, which permits unrestricted use, distribution, and reproduction in any medium, provided the original author and source are credited.

Data Availability Statement: All relevant data are within the paper and its Supporting Information files.

Funding: This work was supported by grants from the National Science Foundation (IBN-05384) and National Institutes of Health (R01-EY014619 and R01-EY024941) to WRJ. The funders had no role in study design, data collection and analysis, decision to publish, or preparation of the manuscript.

Competing Interests: The authors have declared that no competing interests exist.

Abstract

The sclera is the tough outer covering of the eye that provides structural support and helps maintain intraocular pressure. In some fishes, reptiles, and birds, the sclera is reinforced with an additional ring of hyaline cartilage or bone that forms from scleral ossicles. Currently, the evolutionary and genetic basis of scleral ossification is poorly understood, especially in teleost fishes. We assessed scleral ossification among several groups of the Mexican tetra (*Astyanax mexicanus*), which exhibit both an eyed and eyeless morph. Although eyed *Astyanax* surface fish have bony sclera similar to other teleosts, the ossicles of blind *Astyanax* cavefish generally do not form. We first sampled cavefish from multiple independent populations and used ancestral character state reconstructions to determine how many times scleral ossification has been lost. We then confirmed these results by assessing complementation of scleral ossification among the F_1 hybrid progeny of two cavefish populations. Finally, we quantified the number of scleral ossicles present among the F_2 hybrid progeny of a cross between surface fish and cavefish, and used this information to identify quantitative trait loci (QTL) responsible for this trait. Our results indicate that the loss of scleral ossification is common–but not ubiquitous–among *Astyanax* cavefish, and that this trait has been convergently lost at least three times. The presence of wild-type, ossified sclera among the F_1 hybrid progeny of a cross between different cavefish populations confirms the convergent evolution of this trait. However, a strongly skewed distribution of scleral ossicles found among surface fish x cavefish F_2 hybrids suggests that scleral ossification is a threshold trait with a complex genetic basis. Quantitative genetic mapping identified a single QTL for scleral ossification on *Astyanax* linkage group 1. We estimate that the threshold for this trait is likely determined by at least three genetic factors which may control the severity and onset of lens degeneration in cavefishes. We conclude that complex evolutionary and genetic patterns underlie the loss of scleral ossification in *Astyanax* cavefish.

Abbreviations: QTL, quantitative trait loci; CF, cavefish; SF, surface fish; LOD, logarithm of the odds; RPE, retinal pigment epithelium.

Introduction

The Vertebrate Sclera and Scleral Ossification

The vertebrate sclera is the tough outer covering of the eye that provides structural support to the eye and helps maintain intraocular pressure. In humans and other mammals, the sclera is made up of a thick layer of fibrous collagen [1]; however, in birds, reptiles, and fishes, the sclera is made up of a cartilaginous cup that may be reinforced with bony elements called scleral ossicles (reviewed in [2]). Although humans and other mammals lack a cartilaginous sclera and scleral ossicles, they can still develop atavistic ossifications similar to those found in birds and fishes following cancer or ocular trauma [3–5]. Although the sclera and its ossifications are critical to maintaining the normal shape and function of the eye in non-mammalian vertebrates, the genetic mechanisms regulating the development of this tissue have not been studied in detail.

Most of our understanding of scleral development and ossification comes from chicks (reviewed in [6]). In chicks, the scleral cartilage arises from neural crest cells that migrate to the optic cup and are induced to undergo chondrogenesis by the retinal pigment epithelium (RPE) [7]. Underlying scleral papillae then induce the formation of the bony ossicles via one or more diffusible factors [8]. The developmental signaling genes *sonic hedgehog* (*shh*; OMIM: 600725), *indian hedgehog* (*ihh*; OMIM: 600726), and *bone morphogenic protein 2* (*bmp2*; OMIM: 112261) have all been implicated [9]. Following their induction, the scleral ossicles then grow over a period of several days via intramembranous ossification [6]. Several factors have been shown to influence scleral ossicle formation in chicks, including environmental variation, genetic variation [10], and growth rate [11].

More recent research, lead primarily by Franz-Odendaal and colleagues, has focused on the scleral cartilage and ossicles of teleost fish (e.g., [12]). Like chicks, teleosts also possess scleral cartilage and bony ossicles derived from neural crest cells [13]; however, unlike the scleras of birds and reptiles, the scleral ossifications of teleosts form through endochondral ossification [14]. As a result of this and other distinct developmental origins, the scleral elements of teleosts are considered non-homologous to the scleral elements of birds and other tetrapods [6,15]. Furthermore, scleral ossification is evolutionarily labile among teleosts (reviewed in [12]). The ancestral condition appears to be the presence of four ossicles [16], although approximately half of the teleost species so far surveyed (265/547) lack any scleral ossifications altogether [12]. The remaining species exhibit either one (11/457) or two (271/547) scleral ossicles as the result of numerous independent gains and losses [12]. The genetic, developmental, and evolutionary factors responsible for the diversity of scleral ossicle number in teleosts are currently unknown, although adaptation to different depths may play a role [12].

The Astyanax Model System

The teleost fish *Astyanax mexicanus*, or the Mexican tetra, is a natural model system for the study of eye development, including scleral ossification [14,17,18]. These fishes are found in both eyed surface-dwelling (SF) and eyeless cave-dwelling (CF) forms [19]. Like most other Characiform fishes so far surveyed, *Astyanax* surface fish have two scleral ossicles, which appears to be the ancestral state for this group [12,14]. Scleral cartilage is found in both SF and CF morphs, and is induced within the first three days of development, presumably by the RPE [18]. During this time, scleral ossicles are also induced in SF; however, in CF, the scleral ossicles generally do not form [18,20]. Starting at approximately one month of age and continuing for the next two years, the scleral ossicles of SF elongate via unilateral periskeletal ossification and fuse to form a continuous bony ring that eventually covers 90% or more of the circumference

of the eye, while the sclera of CF remain cartilaginous, stop growing, and eventually form a cyst around the degenerating eye [14,20].

The presence/absence of scleral ossicles in *Astyanax* appears to have a genetic basis. SF and CF inherit the presence/absence of scleral ossicles with fidelity from generation to generation [20], and the F_1 hybrid progeny of SF and CF exhibit scleral ossicles [21], suggesting that loss of ossicles is recessive. On the other hand, there also appears to be strong indirect evidence that the lens influences scleral ossicle formation in *Astyanax*, and possibly other teleosts as well. For example, the loss of scleral ossicles is coupled with the degeneration of the embryonic lens in CF, and when tissues from the optic vesicle or lens of SF are transplanted into developing CF, the CF form ossicles [20]. Conversely, the experimental removal of the lens leads to the loss of one or both scleral ossicles in SF depending on the timing of lens ablation. [18]. Lens removal at 1 day post fertilization (dpf) results in the complete loss of the scleral ossicles, while removal at 2 or 3 dpf results in the loss of only one scleral ossicle, and removal at 4 dpf has no effect [18]. Since the earliest stages of scleral development include a simple cartilaginous cup and ring, the evolutionary loss of scleral ossicles in CF may be an example of paedomorphy [14].

Given the role of *Astyanax* as a model system for ocular development and evolutionary genetics, the intraspecific variation in scleral ossicle number observed in this species makes it ideally suited to elucidate the evolutionary and genetic factors influencing scleral ossification in teleosts and other vertebrates. But, despite the wealth of recent research on scleral ossicle development in *Astyanax* described above (e.g., [14,18,20,21]), several important questions remain. For example, the distribution of scleral ossification among different CF populations is currently unknown. To date, scleral ossicles have not been observed among members of the most commonly studied CF population from Pachón cave [20] (but see [22]), but they have been observed among an independently evolved population of *Astyanax* CF from Tinaja cave [18]. At least 27 other CF populations are also found in northeastern Mexico, but these have yet to be sampled for scleral ossification. Whether or not the loss of scleral ossification has evolved independently among these different populations, as has been widely inferred for teleosts in general, is therefore a mystery. Additionally, the number and location of mutations responsible for the loss of scleral ossicles are also unknown. The binary presence/absence of scleral ossicles in surface fish and cavefish suggests that this trait may have a simple genetic basis, but extensive studies of the inheritance of scleral ossicle number have not been performed. Fortunately, the interfertility of *Astyanax* surface fish and cavefish provides us the opportunity to dissect this trait via quantitative trait locus (QTL) mapping. QTL mapping employs the statistical analysis of phenotypic and genotypic distributions in hybrids to identify regions of the genome responsible for variation in some trait of interest [23]. This strategy has already proven successful in identifying a host of QTL and mutations responsible for evolutionary changes affecting eye and pigmentation development in *Astyanax* (e.g., [24–26]). QTL mapping could also provide support for the hypothesis that the lens influences scleral ossification if QTL for scleral ossification co-map to those for eye or lens size.

In the present study we assess three aspects of scleral ossification in *Astyanax*. First, we assess the distribution of scleral ossicles and ossification among different CF lineages by sampling seven different populations, including several from independently evolved "Old" and "Young" lineages [27]. Second, we use ancestral character state reconstructions and a complementation cross to determine how many times scleral ossification may have been lost among these lineages. Third, we use an F_2 intercross of *Astyanax* SF and CF to elucidate the genetic basis of scleral ossicle number and identify QTL for scleral ossification. The results of this study suggests that the loss of scleral ossification has evolved convergently at least three times among *Astyanax* cavefish; however, the skewed inheritance of this trait in SF x CF F_2 indicates

that this seemingly simple phenotype is the result of an underlying genetic threshold with a complex genetic basis.

Materials and Methods

Ethics Statement

This study was approved by the University of Maryland, College Park Institutional Animal Care and Use Committee (IACUC Protocol R-12-53 to WRJ). All fish were euthanized using a lethal dose of 250 mg/L buffered MS-222 following our approved UMCP IACUC protocol.

Sampling

For our first two analyses, we examined scleral ossification in multiple *Astyanax* cavefish populations and their hybrids in order to assess the extent of scleral ossification and its genetic basis in this group. For the population analysis, we selected several members from the "Old" cavefish lineage–Chica (n = 3), Curva (n = 2), Sabinos (n = 3), Pachón (n = 3), and Tinaja (n = 4) caves–as well as two members from the "Young" cavefish lineage–Molino (n = 1) and Rio Subterráneo (n = 2) (Fig 1). For our complementation analysis, we crossed fish from two independently derived "Old" cave populations, Pachón and Sabinos, to assess complementation of scleral ossification among their F_1 hybrid progeny (n = 4) (Fig 2). For both analyses, all fish were approximately 3–5 years old, and should have completed scleral ossification. Sclera ossification was examined in both eyes (or eye orbits) and no variation within individuals was noted.

For our third analysis, we examined scleral ossification among the F_2 hybrid progeny (n = 196) resulting from a cross of *Astyanax* SF from Texas and CF from Pachón cave in order to identify QTL that contribute to scleral ossification. The details of this cross and the resulting genetic map have been described elsewhere [28,29]. The sampled fish fell into three age categories: 2, 4.5, and 5 years old. Prior to sampling, fish were fixed with either 4% paraformaldehyde dissolved in 1x phosphate-buffered saline (PBS) or 100% methanol. Depending on their mode of fixation, the specimens were then stored for 1–2 years in either 1x PBS at 4°C or 100% methanol at -80°C.

Staining for Scleral Ossification

To assess the extent of scleral ossification in all three analyses, we stained fish for bone and cartilage following standard protocols using alizarin red and alcian blue [30]. For the population and complementation samples, we de-scaled and stained whole fish for 12–48 hours. To better visualize the alcian blue stain, we bleached the pigment from each fish by bathing them in a solution of 3% hydrogen peroxide for 12 hours. In most cases, the condition of the sclera in adult cavefish was impossible to accurately determine in stained specimens because of the opacity of adipose tissue in the orbit. Thus, orbital tissue was dissected from each stained individual and restained with alizarin red and alcian blue to accurately determine the condition of the sclera.

For the quantitative genetic samples, we stained only the left eye from each fish. We dissected the left eye using micro-dissecting scissors, working carefully to avoid piercing the eye or damaging the scleral ring. Once removed from the head, we re-fixed all eyes in 4% paraformaldehyde for 2 hours at room temperature. After dissecting the eyes, we stained them all at once in three 96-well boxes used to hold 1 mL pipette tips. To keep track of the samples, we placed each eye in an individual staining cradle created from a 1 mL pipette tip. We cut the nozzle from each pipette tip to the 200 μl mark, heated the sheared end under a Bunsen burner

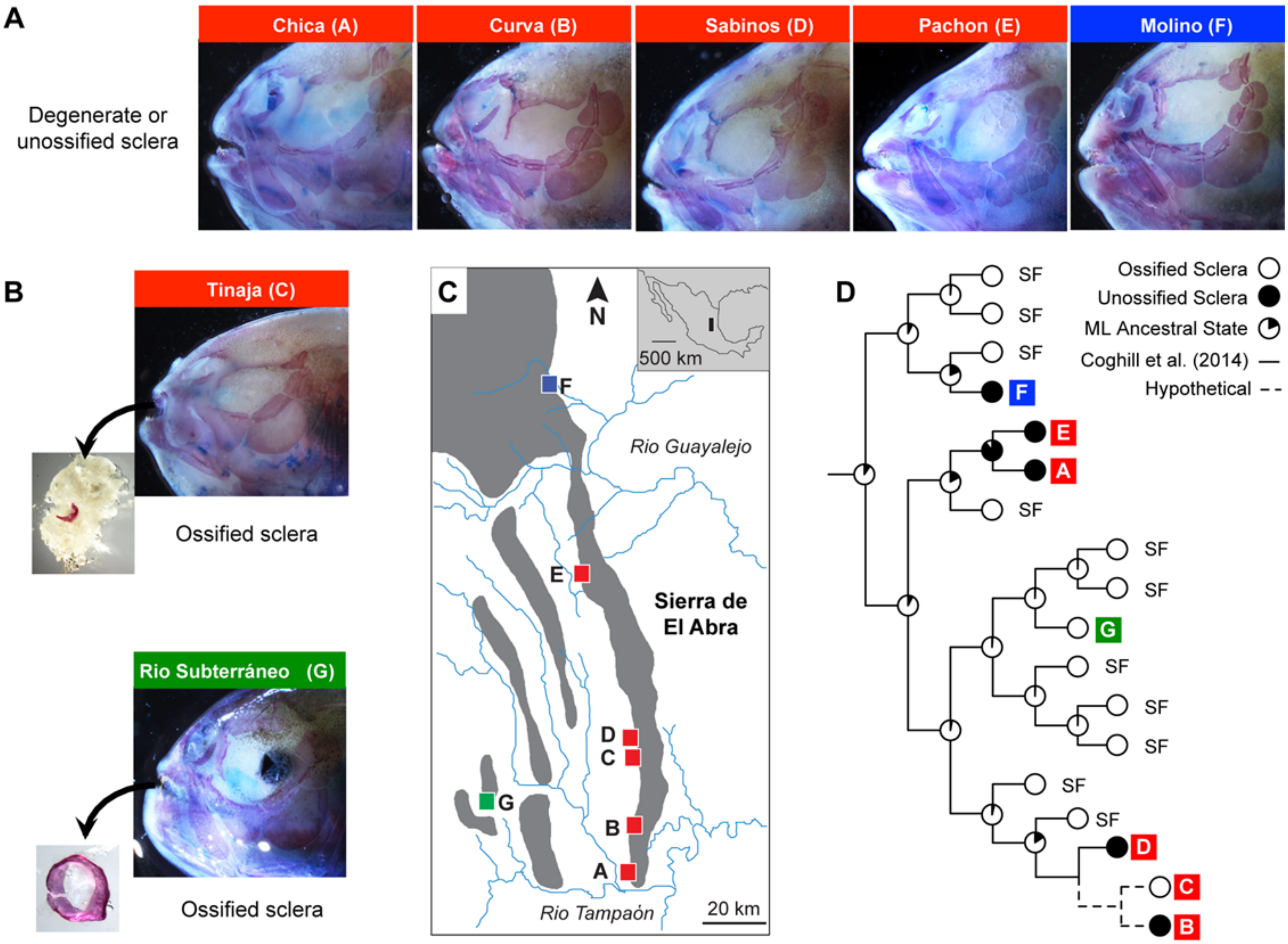

Fig 1. Variation in scleral ossification among eyeless cave populations of the Mexican tetra, *Astyanax mexicanus*. (A) Five populations exhibit degenerate or unossified sclera. Of these, four are members of the evolutionarily "Old" El Abra cavefishes (Chica, Curva, Sabinos, and Pachón; shown in red), and one is a member of the evolutionarily "Young" Guatemalan cavefishes (Molino; shown in blue). **(B)** Two populations exhibit wholly or partially ossified sclera. One is a member of the "Old" El Abra cavefishes (Tinaja) and another is a member of the additional lineage of "Young" cavefishes, the Micos cavefishes (Rio Subterráneo; shown in green). **(C)** The biogeographical distribution of the populations mentioned in A and B, along with 22 other known populations. Red = El Abra (A–E), Blue = Guatemalan (F), Green = Micos (G). **(D)** Ancestral character state reconstruction of scleral ossification using a recent molecular phylogeny of 11 surface fish populations and 5 of 7 cavefish populations used in this study [35]. The hypothetical positions of the remaining two populations are shown with a dashed line. Pie charts on internal nodes illustrate the ML support for ossified (white) vs. unossified (black) sclera at each ancestral position. Assuming that all surface fish populations exhibit ossified sclera, the ancestral character state reconstructions suggest that scleral ossification has been lost at least three times among the various cavefish populations.

doi:10.1371/journal.pone.0142208.g001

for 3–5 seconds until just soft, and then fixed a piece of nylon-webbing to the tip to allow stain to enter the cradle. After making these cradles, we placed one eye inside each individually-labeled pipette tip, placed each tip into the 96-well frame, and then placed the whole frame back inside the pipette tip box for staining. We stored all stained sclera in a solution of 1x PBS and 0.5% sodium azide at 4°C until further imaging. All sclera were imaged within one week of staining.

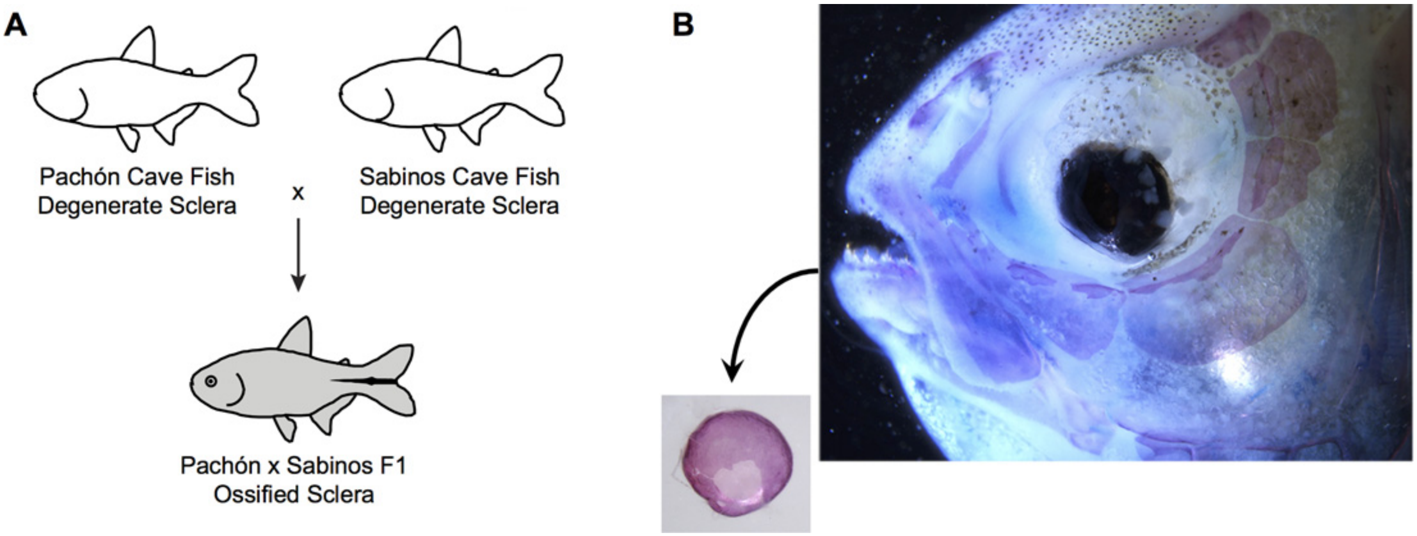

Fig 2. Restoration of scleral ossification following complementation between two different cavefish populations. (A) Diagram shows the results of crossing Pachón and Sabinos cavefishes. Diagram modified from Wilkens [54]. **(B)** A small ossified sclera develops in the F_1 progeny of a Pachón × Sabinos cross with restored eyes.

doi:10.1371/journal.pone.0142208.g002

Quantification of Scleral Ossification

For the population and complementation samples, we qualitatively assessed scleral ossification by dissecting the stained specimens and visually noting the presence/absence of ossified elements within the sclera. The presence of any ossifications within the sclera was interpreted as evidence of scleral ossification. For the quantitative genetic samples, we measured three aspects of scleral ossification: (1) the number of scleral ossicles present within the sclera (either 0, 1, or 2); (2) the proportion of the circumference of the eye occupied by scleral ossicles, following a modified form of the method used by Franz-Odendaal et al. [31]; and (3) the width of the scleral ring divided by the total diameter of the eye. To estimate the number of ossicles, we simply counted the number of continuous ossified regions on the anterior and posterior portions of the eye. When a single continuous ring was found on both portions of the eye, this was counted as two distinct (though fused) ossicles. To estimate the proportion of the circumference of the eye occupied by scleral ossicles, we positioned each F_2 eye cornea-down in a solution of 1x PBS and photographed the coronal (back) portion of each eye using a Zeiss Discovery v20 Stereomicroscope. This position gave us the best estimate of the degree of ossification surrounding the entire eye. After photographing the eye, we used the image analysis program ImageJ v 1.47t [32,33] to first estimate the total circumference of the eye using the formula $C_{total} = \pi\times$ eye diameter. We then used the broken segment line tool of ImageJ to measure the circumference of the eye that was occupied by bone (C_{bone}). Dividing C_{bone} by C_{total} provides the proportion of the circumference of the eye occupied by scleral ossicles. In cases where the sclera was mostly ossified, we simply measured the circumference of the eye that was unossified, and subtracted this from C_{total} before dividing to get the proportion of the circumference of the eye occupied by scleral ossicles. Finally, to estimate the relative width of the scleral ring, we used the line segment tool of ImageJ to measure the width of the sclera ring in the ventral portion of the eye. We then divided the width of the ring by the total diameter of the eye.

Ancestral State Reconstruction and Complementation Test

To determine the number of losses of scleral ossification among the different cavefish populations sampled, we traced the evolution of the qualitative presence/absence of scleral ossification among *Astyanax* CF and SF populations via Maximum Likelihood ancestral character state reconstruction [34]. Our reconstruction utilized a robust molecular phylogeny of 11 *Astyanax mexicanus* SF populations and five of the seven CF populations used in this study (all populations except Curva and Tinaja) [35]. For the sake of continuity, we added the two remaining cavefish populations to the phylogeny based on their hypothetical positions given trees produced by mitochondrial ND2 sequences and six nuclear microsatellite loci [36,37]; however, we did not include these populations in the reconstruction. We also note that we did *not* assess sclera ossification in any of the surface fish populations included in this phylogeny, but instead assumed that all exhibit ossified sclera. We feel this assumption is justified since all SF so far examined possess scleral ossicles, and the development of these structures in SF have been described in detail [14,18,20]. Additionally, like *Astyanax* surface fish, most other Characiform fishes also posses two scleral ossicles, as do approximately half of all teleost fish species so far surveyed [14,38]. Thus, it seems reasonable to assume that the presence of two scleral ossicles represents the ancestral state for *Astyanax mexicanus*. We reconstructed ancestral states for scleral ossification via Maximum Likelihood assuming unordered characters with an equal probability of transition between ossification and loss of ossification. We performed this reconstruction with the program Mesquite v2.75 [39].

In addition to our test of independent evolution via ancestral state reconstruction, we also tested this hypothesis via genetic complementation. We assessed scleral ossification in the F1 hybrid progeny of two independently derived cavefish populations as described above. Complementation occurs when the hybrids of two independently derived mutant strains exhibit the wild-type condition (in this case, the presence of scleral ossification), which indicates that the mutations responsible for the mutant phenotype are different in each strain. The presence of ossified sclera in any of the F_1 progeny would provide evidence of complementation and suggest that different mutations–and, hence, convergent evolution–are responsible for the loss of scleral ossification in these two CF populations. A similar strategy has previously been used to demonstrate convergence in eye loss [22] and pigmentation [24] among different *Astyanax* cavefish.

Quantitative Trait Locus (QTL) Mapping

Our analysis of quantitative trait loci (QTL) contributing to scleral ossification followed our previously published protocols [28,29]. Briefly, we started with a genetic linkage map of 698 microsatellite and SNP markers [29]. We scanned this genetic map for QTL associated with the our three measures of scleral ossification using the function *stepwiseqtl* in the program R/qtl [23,40]. We calculated the logarithm of the odds (LOD) of association between each measure of scleral ossification and the genotypes at each genetic marker using Haley-Knott regression. Since scleral ossification has been shown to increase with age [14], we included both the age and standard length of each fish as additional covariates. We assessed the statistical significance of the resulting LOD scores by calculating the 95th percentile of genome-wide maximum penalized LOD scores for each phenotype using 1,000 random permutations of the genotypic and phenotypic data using the R/qtl function *scantwo*. These permutations were performed in two stratified groups. One group included 141 F_2 that were genotyped at 235 microsatellite markers [28] and the other included 56 F_2 that were genotyped at an additional 463 SNPs [29]. We defined the confidence intervals for the position of any QTL using 95% Bayesian credible

intervals expanded to the nearest genotyped marker. The phenotypic and genotypic data used for all QTL analyses are available in S1 and S3 Tables.

Results

Convergent Loss of Scleral Ossification in Astyanax Cavefish

For our first analysis, we sampled seven different populations of cavefish, including several members of the independently-derived "Old" and "Young" lineages, and qualitatively assessed them for the presence/absence of scleral ossification (Fig 1A–1C). The majority of the individuals sampled possessed no observable scleral ossification, either upon staining or following dissection. Cavefish populations with such degenerate sclera included Pachón, Sabinos, Curva, and Chica ("Old" lineage) as well as Molino ("Young" lineage) (Fig 1A). However, we did find two populations with ossified sclera: Tinaja CF (members of the "Old" lineage) had a partially degenerate scleral ring that remained ossified, as reported previously by Dufton et al. [18]; and Rio Subterráneo CF (members of the "Young" lineage) had a small scleral ring that was complete and entirely ossified (Fig 1B). These differences suggest that the genetic and environmental factors that control scleral ossification vary among different cavefish populations, and may have evolved multiple times among several independently-derived populations. Reconstruction of ancestral character states for the presence/absence of scleral ossification among five of these populations supports this interpretation, indicating that scleral ossification has been independently lost at least three times among *Astyanax* cavefishes (Fig 1D): once among the lineage including the "Young" Molino population (Guatemalan cavefishes); a second time among the lineage containing several "Old" populations, including Pachón and Chica (El Abra cavefishes); and a third time among a lineage containing Sabinos and additional "Old" cavefishes including Curva (Fig 1D). In contrast, the ancestral presence of scleral ossicles seems to have been preserved in two populations: Tinaja, a member of an "Old" (El Abra) cavefish lineage, and Rio Subterráneo, a member of the "Young" (Micos) lineage. Thus, scleral ossification appears to be as evolutionarily labile among populations of cavefish as it is among teleosts in general, and Characiforms especially [12,38].

Complementation Confirms Convergent Loss of Scleral Ossification

For our second analysis, we sought to confirm the results of our ancestral character state reconstruction by demonstrating the complementation of scleral ossification among the F_1 hybrid offspring of two independently-derived cavefish populations from Pachón and Sabinos caves [22,41] (Fig 2A). Half of the F_1 progeny examined (2/4) exhibited small, pigmented eyes with ossified sclera on both sides of their head (Fig 2B). Although our sample size is small, the results of this experiment clearly demonstrate genetic complementation of scleral ossification and experimentally confirm the convergent loss of sclera ossification among different *Astyanax* cavefish populations.

Skewed Distribution of Scleral Ossicles Among F_2 hybrids

For our third analysis, we quantified scleral ossification from the left eyes of *Astyanax* SF × Pachón CF F_2 hybrids (n = 196). We observed a range of phenotypes, from eyes with no scleral ossicles to those with two fused ossicles that occupied the entire circumference of the eye (Fig 3A and 3B). Surprisingly, however, this diversity was not normally distributed among individuals with 0, 1, and 2 scleral ossicles, as would be expected for a trait with an additive genetic basis. Instead, the vast majority (181/196) of the F_2 hybrids possessed two elongated scleral ossicles that occupied $\geq$90% of the circumference of the eye (Fig 3C). Only 4 hybrids

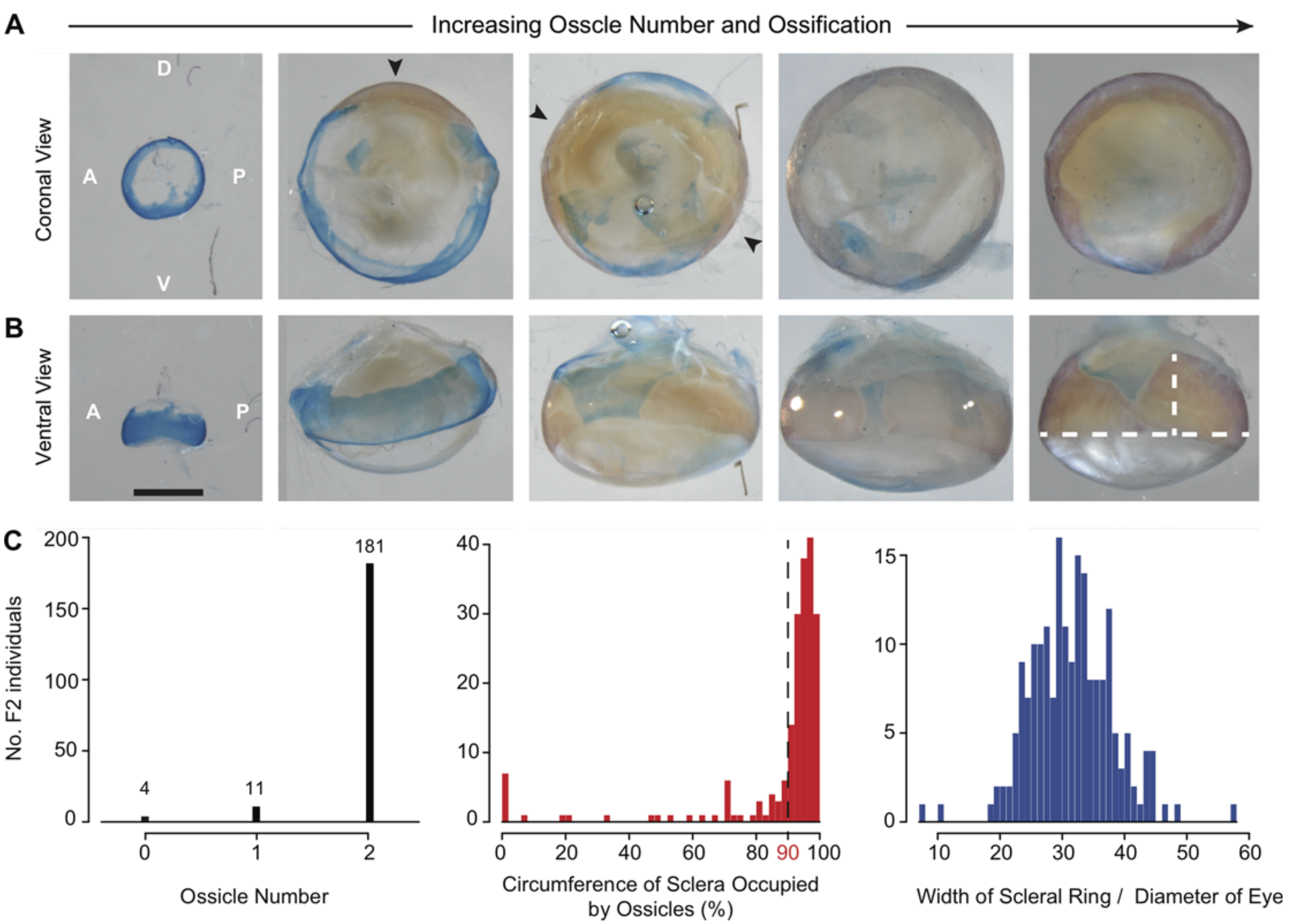

Fig 3. Limited phenotypic variation in scleral ossification among the progeny of a SF x CF F_2 hybrid cross. (A) Illustrative examples of reduced and normal scleral ossicle formation among 196 hybrid F_2 progeny. Individual scleral ossicles are stained red with alizarin and highlighted with black arrows. **(B)** A ventral view of the same eyes as in **(A)**. The length of the scleral ossicles varied dramatically, with some that occupy the entire circumference of the eye. White bars on the last image indicate where the width of the scleral ring and diameter of the eye were measured. **(C)** Distribution of the scleral phenotypes measured in this study. Both the number of scleral ossicles and the circumference of the sclera occupied by ossicles were highly skewed towards the wild-type phenotype of two ossicles that occupy >90% of the eye. Dotted line in the second panel indicates the threshold (90%) used to denote wild-type versus reduced scleral ossicles.

doi:10.1371/journal.pone.0142208.g003

recapitulated the full cavefish phenotype of sclera with zero ossicles, while only slightly more (n = 11) possessed just one ossicle instead of two (Fig 3C). Of these 11 individuals, the single ossicle was severely reduced, covering, on average, only 28% of the circumference of the eye. Rather than a simple Mendelian trait or even an additive polygenic trait, this phenotypic distribution is strongly indicative of a polygenic threshold, as described by Wright [42] for guinea pig digit number [43]. In contrast, the width of the scleral ring relative to the diameter of the eye exhibited a much more normal distribution. Consistent with a previous report by Franz-Odendaal et al. [14], we found that the proportion of the circumference of the eye occupied by scleral ossicles was positively correlated with both the age (Pearson's $r = 0.220$, df = 195, $t = 3.13$, $P = 0.002$) and overall standard length (Pearson's $r = 0.337$, df = 194, $t = 4.980$, $P < 0.00001$) of the each fish, hence their appropriate use as covariables in this analysis. Unfortunately, the limited variation caused by the skewed distribution of scleral ossicles, as well as

the presence of significant covariables, likely limits our power to detect QTL for scleral ossification in this cross.

In addition to scleral ossification, we also quantified eye diameter, eye area, and pupil area in 115 F_2 in order to test the hypothesis that any QTL for scleral ossification overlap existing QTL for eye or lens size. All three eye phenotypes were normally distributed (results not shown), which is consistent with numerous previous analyses of eye and lens size in *Astyanax* hybrids [22,26,44].

QTL for Scleral Ossification

Following our measurements of scleral ossification, we scanned the *Astyanax* genome for quantitative trait loci (QTL) associated with scleral ossification while controlling for age and standard length as covariates. We initially found no significant QTL for scleral ossification when treated as a continuous trait, although we did find two marginally non-significant QTL at the significance threshold $P < 0.07$ (Fig 4A). The first marginally non-significant QTL, found on *Astyanax* linkage group 1, is associated with the proportion of the circumference of the eye occupied by scleral ossicles (LOD = 3.64, $P = 0.065$; results not shown; see below). At the peak QTL position of 59.5 cM, F_2 hybrids with two CF alleles have a smaller proportion of their eye occupied by scleral ossicles than individuals with one or more SF alleles (LOD = 3.64, $P = 0.065$). This effect is consistent with CF possessing fewer scleral ossicles. The second QTL, on *Astyanax* linkage group 2 from 0.00–78.1 cM, is associated with width of the scleral ring relative to total eye diameter. At the peak QTL position of 0.00 cM, F_2 hybrids with one or more CF alleles have wider scleral rings relative to the total diameter of their eye in the ventral portion of their sclera (LOD = 3.68, $P = 0.064$ (Fig 4). This effect is consistent with CF possessing wider scleral rings relative to the small size of their eyes. Although these marginally non-significant results provide only weak evidence of QTL for scleral ossification, the results become more significant if scleral ossification is treated as a threshold trait (Fig 4). If F_2 individuals wherein >90% of the circumference of the sclera is occupied by scleral ossicles are scored as "wild-type", and individuals wherein <90% of the circumference of the sclera is occupied by scleral ossicles are scored "reduced" (see dotted threshold line in Fig 3C), the LOD of the ossification QTL on LG 1 increases to 3.88, which is significant at P = 0.033 (Fig 4; S3 Table). This result is consistent with both an abundance of CF genotypes among F_2 individuals that exhibit reduced scleral ossification, as well as the recessive inheritance of reduced scleral ossification [21].

In addition to scleral ossification, we also searched for QTL associated with eye and lens size to determine their relationship to any scleral QTL. We identified eight separate QTL for eye diameter, eye area, and pupil area that were each significant at $P < 0.01$. (S1 Fig and S3 Table). Of these eight, three were adjacent to or completely overlapped the QTL found for scleral ossification (black boxes in Fig 4). The first, on LG 1 from 46.3–51.3 cM, is associated with eye area (Fig 4 and S1 Fig). Although this eye-related QTL does not directly overlap the one significant QTL for proportion of the scleral circumference occupied by scleral ossicles, previous studies have identified a QTL for eye size that overlaps this region (white bar on Fig 4; S1 Fig) [44,45]. Similarly, on LG 2, we identified two QTL, one for eye diameter from 0.00–8.93 cM and another for pupil area from 0.00–47.6 cM (black bars, Fig 4; S1 Fig). Previous studies have also identified QTL for eye and lens size in this genomic location [44,45]. These QTL directly overlap the marginally-nonsignificant QTL for the relative width of the sclera ring (Fig 4). Additional eye and lens size QTL were found on LGs 7, 11, 18, and 23 (S1 Fig and S3 Table). Importantly, two of these eye-related QTL–one for eye area on LG 11 and another for eye diameter on LG 18 –have not previously been described in other studies. Although it is possible

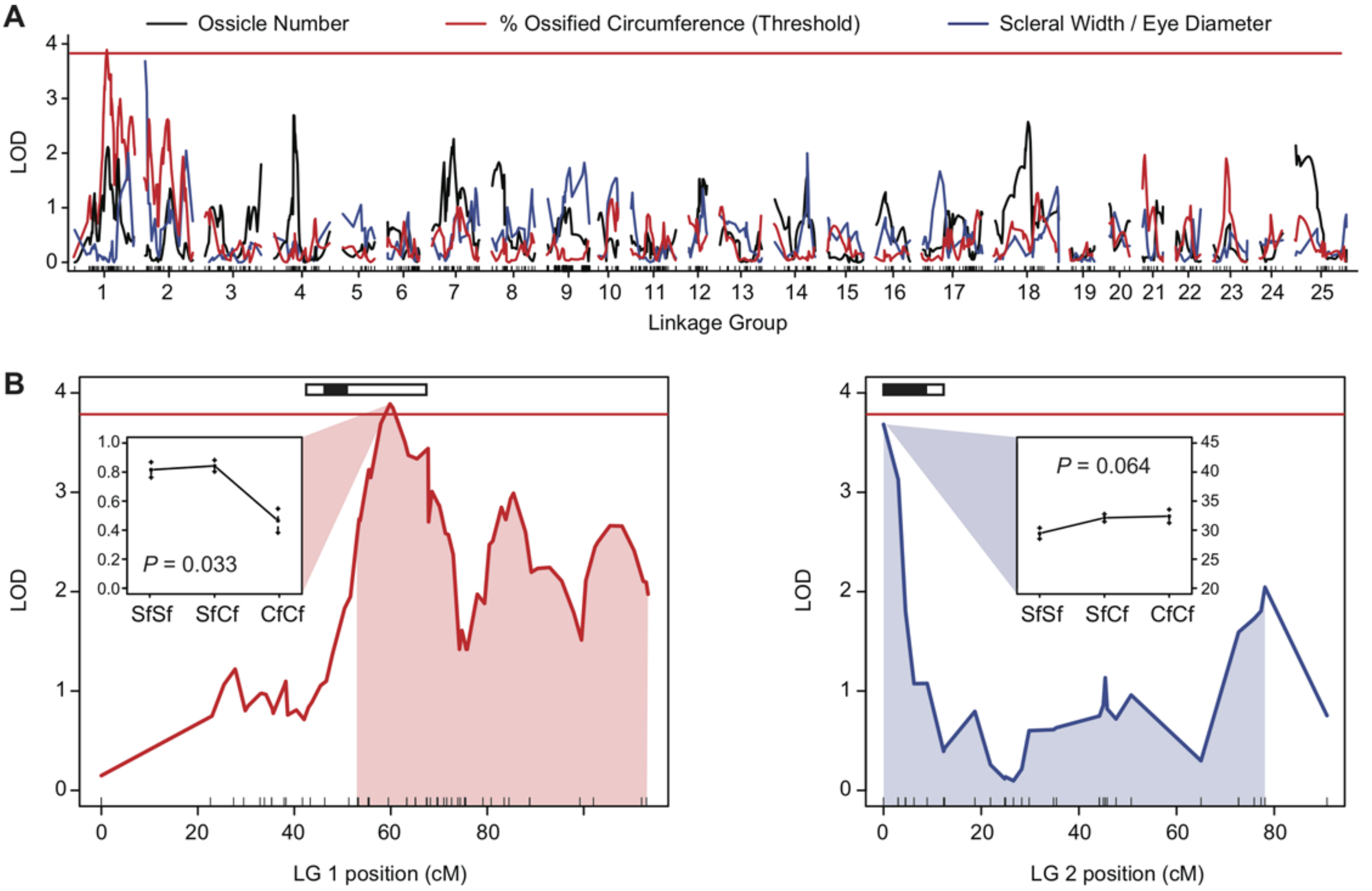

Fig 4. Quantitative trait loci (QTL) for *Astyanax* scleral ossification. (A) Quantitative trait locus (QTL) mapping identified one QTL for the proportion or percent of the circumference of the eye occupied by scleral ossicles on linkage group (LG) 1, and another QTL for the relative width of the sclera on LG 2 that was marginally non-significant at P < 0.07. No QTL was found for ossicle number, though the power of this analysis was likely limited by the small number of F_2 individuals with only 0 or 1 scleral ossicles (see Fig 3). Please refer to O'Quin et al. [29] for the complete genetic linkage map used in this analysis. **(B)** Detailed view of the scleral QTL on LGs 1 and 2. Red line indicates P = 0.05; any value above that threshold is considered statistically significant. Shaded fields indicate the 95% Bayesian confidence interval for the location of the QTL. Inset boxes show the mean phenotypes for each genotypic class at the peak QTL marker. Black and white boxes at the top of the chart indicate the position of eye or lens size QTL found in this (black) or other (white) studies (see also S1 Fig).

doi:10.1371/journal.pone.0142208.g004

that the close association of eye and scleral QTL found on LG 1 and 2 is the result of linkage or even coincidence, this result is at least consistent with the experimentally determined role of lens degeneration in pleiotropically reducing scleral ossification [18].

Discussion

Convergent Loss of Scleral Ossification Among Astyanax Cavefish

Previous reports have found that alternate morphs of the teleost fish *Astyanax mexicanus* differ in their number of scleral ossicles: the eyed surface fish morph exhibits two elongated scleral ossicles that span the circumference of the eye, while, in general, the blind cavefish morph lacks scleral ossicles all together [20]. But, until now, the extent of scleral ossicle reduction and its evolution in different cavefish populations was largely unknown. Based on our ancestral reconstruction of scleral ossification among seven cavefish populations, we conclude that scleral ossification has regressed convergently at least three times among Mexican cavefishes

(Fig 1). Espinasa et al. [46] also reported the loss of scleral ossicles among *Astyanax* cavefish from Granadas cave, which potentially represents a fourth instance of scleral ossicle reduction. But despite the repeated evolutionary loss of scleral ossification, we find that the loss of scleral ossicles is not universal. In particular, we found that individuals from the Tinaja and Rio Subterráneo caves retain scleral ossification despite the fact that both populations still exhibit eye reduction (Fig 1). These results confirm and extend those of Dufton et al. [18], which also found that Tinaja CF exhibit ossified sclera. The discovery that an additional population, Rio Subterráneo, also exhibits wild-type scleral ossification makes sense in light of the history of this population. Rio Subterráneo represents a hybrid population that includes members with both troglomorphic and non-troglomorphic features [19,22]; therefore, it is not surprising that members of this population also possesses complete and even ossified sclera. However, the observation that the loss of scleral ossification is not ubiquitous is still significant because it suggests that eye degeneration may occur without a concomitant loss of scleral ossification.

We then confirmed the results of our ancestral character state reconstruction using a complementation cross of Sabinos and Pachón CF (Fig 2). We observed ossified sclera among the F_1 hybrid progeny of these independently-derived CF lines (Fig 2). To our knowledge, no previous authors have described the presence of even small ossified scleral elements within the eyes of CF from either population, with the exception of Wilkens [22]. Wilkens [22] also performed a complementation cross of Sabinos and Pachón CF and found that sections of eyes taken from the hybrid F_1 had bony scleral elements stained with azan; however, Wilkens [22] also reported that the eyes of Pachón cavefish have bony sclera. This latter result is presented as an aside and is not visible in the figures given, and has not been confirmed by other authors since [20]. Thus, although bony scleral ossicles are found in at least two cavefish populations (Tinaja and Rio Subterráneo; see above), they have not been reported for the two populations analyzed in our cross. Thus, our results provide clear evidence of complementation since we directly stained the sclera for bone and none of the Pachón we sampled possessed scleral ossicles. Although our sample size was small and only half (n = 2/4) of the F_1 hybrids we examined exhibited complementation of scleral ossification, the ossified sclera were observed in both eyes. Complementation analysis has also been used to examine the genetic basis of numerous other traits in *Astyanax*, including eye size, vision, and pigmentation ([22,24,41,47]). Like ours, these studies report a wide range of F1 hybrids that exhibit complementation of wild-type traits following a cross of two mutant cavefish strains. For albinism, a monogenic pigmentation trait, Protas et al. [24] reported that 100% of F_1 hybrids derived from albino Molino and Pachón cavefish exhibit pigmentation. On the other hand, for complex eye and vision related traits, Wilkens [22] reported that as few as 10% of F_1 derived from eyeless Sabinos and Pachón cavefish exhibit larger-than-normal eyes, and Borowsky [41] reported that as few as 1% of F_1 derived from blind Molino and Pachón cavefish exhibit vision. However, in each case, the presence of wild-type, surface fish-like traits among the F_1 hybrid progeny of two independently-derived mutant cavefish strains provides evidence of complementation and, thus, independent evolution. The same principle is true here. The presence of ossified sclera among the F_1 hybrid progeny of Sabinos and Pachón cavefish confirms the complementation of scleral ossification and its independent evolution among different cavefish populations.

The convergent evolution of scleral ossification found here is consistent with the role of *Astyanax* cavefish as paradigmatic examples of repeated evolution. Indeed, comparative analyses have revealed that numerous troglomorphic traits have evolved either convergently or in parallel among different cavefish populations, including pigmentation, eye reduction, foraging behavior, sleep loss, circadian rhythm, and aggressive behavior [24,25,35,36,41,47–50]. In light of such widespread convergence, it would be surprising if the loss of scleral ossification had *not* evolved convergently among at least some members of this group. Furthermore, it seems that

the alteration of scleral or cranial ossification may have evolved independently among other cavefishes as well. For example, scleral ossification is wholly or partially lost among Siluriform cave catfishes from Texas [51] and trench-dwelling cichlids from the Congo river [52]. Hence, the loss of scleral ossification may be a common–though certainly not ubiquitous–feature of eye degeneration in Mexican cave tetras, and possibly other cavefish species as well. Indeed, scleral ossification appears to be as evolutionarily labile among populations of cavefish as it is among teleosts in general, and Characiforms especially [12,38]. Franz-Odendaal [12] argued that the diversity of teleost scleral ossicles may have evolved as an adaptation to increasing depths [12]. Poulson [53] has reviewed the striking similarities between cave and deep-sea environments, including reduced light and productivity, and noted the convergent evolution of numerous traits associated with these unique habitats in cavefish and deep-sea fish. These convergent traits include reduced pigmentation and eyes, as well as expanded non-optic sensory systems such as chemosensory and lateral line receptors [53]. Thus, the results reported here for *Astyanax* may support Franz-Odendaal's [12] hypothesis that the loss of scleral ossicles in teleosts is a response to cave-like environments.

Complex Quantitative Genetics of Scleral Ossification, a Threshold Trait

Previous reports on in the inheritance of scleral ossification suggest that the presence/absence of scleral ossicles has a genetic basis and is inherited as a recessive trait in cavefish [20,21]. Whether or not that genetic basis might be caused by a few or many genes was unknown. The results reported here are the first to examine ossicle number and overall ossicle length in SF x CF F_2 hybrids in order to better dissect the genetic basis of this trait. Surprisingly, we found very limited diversity in scleral ossification among our SF x CF F_2 hybrids (Fig 3). Instead, the vast majority of F_2 exhibited two elongated scleral ossicles that reached around 90% or more of the eye, which is similar to the ancestral surface fish phenotype. Only 7% of the F_2 (15/196) had one or fewer ossicles, and only 2% (4/196) had no ossicles as in most *Astyanax* cavefish (Fig 3). Such a skewed phenotypic distribution suggests that the simple presence/absence of scleral ossicles is not as simple as it may seem. Instead, this phenotypic distribution is indicative of a threshold or quasi-continuous trait [43]. Threshold traits are generally expressed qualitatively (e.g., presence/absence) but their underlying genetic basis is continuous, and the trait will only be expressed in individuals that reach some threshold of underlying genotypic values [43]. Although less common than additively polygenic traits, threshold traits have been described for other troglomorphic traits in *Astyanax*, including oxygen consumption, feeding angle, melanophore distribution, and eye size (reviewed in Wilkens [54]). Coupled with the presence of two significant covariables (age and overall standard length), the limited phenotypic diversity afforded by this unusual mode of inheritance likely reduced our power to detect QTL for scleral ossification. Indeed, we found only two marginally non-significant QTL for scleral ossification ($P < 0.07$) when ossicle number and degree of scleral ossification were treated as quantitative traits (Fig 4), although we found that the significance increased to $P = 0.033$ when scleral ossification was treated as a binary threshold trait (Fig 4). These results demonstrate that the genetics of scleral ossification may be more complex then the binary phenotypes of cavefish and surface fish would initially suggest.

What might be the underlying genetic cause of a threshold for scleral ossicle development in *Astyanax* cavefish? The results of Yamamoto et al. [20] and Dufton et al. [18] both suggest that lens degeneration and its onset may explain the loss of scleral ossicles in *Astyanax*, including the presence of a phenotypic and genotypic threshold. Yamamoto et al. [20] found that transplantation of the surface fish lens into cavefish eyes at 1 day post fertilization (dpf) could restore scleral ossicles in cavefish, implicating cavefish lens degeneration in the loss of scleral ossicles. Dufton et al. [18] extended these results by experimentally ablating the lenses of

surface fish at 1, 2, 3, and 4 dpf and assessing the impact on scleral ossicle formation. When lens ablation occurred at 1 dpf (the same stage used in Yamamoto et al. [20]), most individuals failed to develop scleral ossicles, or else developed scleral ossicles that were reduced in size. However, the results were reversed when lens ablation was performed at 4 dpf: most individuals developed sclera ossicles, and these ossicles were predominantly normal in size. Combined with their results for intermediate stages, Dufton et al. [18] found that the degree of scleral ossicle reduction was highly dependent on the timing of lens removal, with a timing threshold set by the window for scleral ossicle induction during the first 1–2 days of development.

Viewed in light of the genetics of lens degeneration, the threshold for scleral ossicle development becomes even clearer. Here and in other studies, researchers have identified 1–4 QTL responsible for lens size and degeneration, which all likely represent a minimum number of QTL [44,45] (S1 Fig; S1 Table). Following existing evidence and the hypothesis of a genotypic threshold for scleral ossification, we may posit then that (1) at each QTL for lens degeneration, the alleles found in cavefish reduce lens size, as has generally been shown for all lens and all but one eye QTL [44]; (2) cavefish populations are likely fixed for cavefish alleles at all responsible QTL, which would be consistent with the reduced genetic diversity found in this group [37]; and (3) cavefish alleles are likely required at all responsible QTL to produce the dramatic and early loss of lens function required to inhibit scleral ossicle formation at 1 dpf. Under this scenario, we can estimate the number of loci required to inhibit scleral ossicle development by comparing the observed ratio of F_2 progeny with (1+) and without (0) sceral ossicles to the ratios expected for a trait controlled by one or more loci. Our observed ratio of 48:1 (192:4) is consistent with a genotypic threshold caused by three loci, but not with thresholds caused by one, two, or four loci (Table 1). A similar result is obtained if the variance of the F_2 is used to estimate the minimum number of genetic factors underlying scleral ossicle number, n_e, assuming that purebred surface fish and cavefish exhibit negligible variation in scleral ossicle number (Castle-Wright estimator $n_e = 3.88$) [42,43]. Indeed, under a scenario where recessive alleles are required at three loci to inhibit scleral ossicle development, SF x CF F_2 hybrids are only expected to exhibit scleral ossicle loss in 3/196 individuals, or $0.25^3 = 1.56\%$ of the F2 population. This expectation is clearly met in our study, as we observed scleral ossicle loss in only 4/196 = 2.04% of the F_2 population ($\chi^2 = 0.338$, df = 1, $P > 0.05$; see Table 1). The small deviation

Table 1. Chi-square analyses of observed and expected ratios of F_2 progeny with (1+) and without (0) scleral ossicles due to a genotypic threshold at 1–4 loci.

No. Loci	Expected Phenotypic Ratio	Expected Number of F2 Progeny & Genotypes		χ^2	P-value
		1+ ossicles	0 ossicles		
Observed	48:1	192	4	NA	NA
		?	?		
1	3:1	147	49	55.10	< 0.0001
		A-	aa		
2	15:1	184	12	5.681	0.0171
		A- —	aabb		
3	63:1	193	3	0.146	0.7029
		A——	aabbcc		
4	255:1	195	1	9.046	0.0026
		A———	aabbccdd		

F_2 progeny without scleral ossicles are expected to be recessive at all responsible loci (A–D), while those with 1+ scleral ossicles are expected to have at least one dominant allele at any of the responsible loci, but which is represented here by A.

doi:10.1371/journal.pone.0142208.t001

between the observed and expected values shown here may be attributable to environmental variation. However, we note that this result only accounts for the genotypic threshold responsible for the complete loss of scleral ossicles; a second threshold responsible for the development of at least one ossicle also exists, but is not accounted for by this simple model. Future studies can use a larger F_2 family to dissect this phenotype more fully. But, in either case, these results suggest that, although the loss of scleral ossification may be a binary trait in cavefish and surface fish, individuals must be fixed for regressive cavefish alleles at three loci in order to inhibit scleral ossicle development. Although this requirement may be easily met in natural populations of surface fish and cavefish alternately fixed at multiple alleles for lens degeneration, only a small fraction of individuals in hybrid or admixed populations are expected to meet this requirement. Such a genotypic threshold not only explains the low occurrence of scleral ossicle loss among our F_2, but it may also explain our finding that the loss of scleral ossification is not ubiquitous among cavefishes. Indeed, previous authors have noted that cavefish populations can vary in the onset and severity of lens degeneration [54], and several populations are the result of recent or ongoing hybridization with surface fish, including those at Chica and Rio Subterráneo caves [19,22]. Given this phenotypic and genotypic diversity, it should be unsurprising that scleral ossicles are still found among phylogenetically young or admixed cavefish groups.

Implications for Future Studies

The results of this study have several implications for future studies of scleral ossification in *Astyanax* and other teleosts. For example, since the results of Yamamoto et al. [20] and Dufton et al. [18] demonstrate that scleral ossification is dependent on the optic cup and lens [18,20], one might hypothesize that at least some of the QTL for scleral ossification co-map with QTL for eye or lens degeneration. Our results provide only weak support for this hypothesis. Although we did identify QTL for lens and eye size on the same linkage groups as our scleral QTL (Fig 4; S1 Fig), only the QTL for lens and eye size on LG 2 overlap the scleral QTL directly (although other studies have identified QTL for eye size that overlap the scleral QTL on LG 1 [28,44,45]) (Fig 4). The correlation between QTL for scleral ossification and those for eye or lens size is significant since it is consistent with previous experimental results which show that the lens removal influences scleral ossification in *Astyanax* [18,20], and also suggests a possible role for pleiotropy in the loss of scleral ossification. However, the role of pleiotropy versus a direct genetic basis for scleral ossicle formation is still not clear. Although Yamamoto et al. [20] found that transplanting surface fish lenses at 1 dpf could rescue scleral ossification in cavefish, the opposite experiment did not produce comparable results. When the lenses of cavefish were transplanted into the eyes of surface fish, the scleras of surface fish still ossified normally, despite the early timing of lens transplantation and accompanying eye reduction. This result suggests that different response of the sclera to lens removal in surface fish and cavefish could still have its own genetic basis. Additionally, in light of the complex genetic basis of scleral ossification, improvements to our experimental design may increase our power to detect QTL for this trait in the future. Sample size is an obvious start, since we expect only a small number of F_2 hybrids to exhibit the cavefish phenotype of reduced scleral ossicles. However, a better design would be a backcross or testcross, which would greatly increase the number of mutant progeny for analysis, thus improving the power of future studies to detect QTL for ossicle development. Additionally, our study assessed scleral ossification in adult fish ranging in age from 2–5 years old. Given that age is significantly associated with degree of scleral ossification in teleosts (see Results; [14]), future studies may limit any variation in scleral ossification due to size or age by assessing fish of similar age during the first month of development when the

scleral ossicles initially form. If possible, it would also be desirable to control for the onset of lens degeneration by using individuals with similar sized lenses [14,18].

Conclusions

We dissected the evolution and quantitative genetic basis of scleral ossification in the Mexican tetra, *Astyanax mexicanus*, which exhibits both eyed surface and eyeless cavefish morphs. We found that the scleral ossicles have been reduced convergently among several eyeless cavefish populations, although their loss is not ubiquitous. Our attempt to identify quantitative trait loci for scleral ossicle number and percent ossification successfully identified one QTL, although we found that the inheritance of scleral ossification to be much more complex than this seemingly binary trait might suggest. Instead multiple loci responsible for the onset of lens degeneration may generate a genetic threshold responsible for scleral ossicle reduction. Future work will aim to quantify scleral ossification earlier while potentially controlling for confounding traits like age and the onset of lens degeneration. We will also identify candidate genes and mutations within our QTL to assess their impact, if any, on *Astyanax* scleral ossicle development. Thus, *Astyanax* may serve as a useful evolutionary model for understanding scleral ossification formation in teleosts and other vertebrates, including humans.

Supporting Information

S1 Fig. Quantitative trait loci (QTL) for *Astyanax* eye and lens size. Detailed view of the eye or pupil size QTL on *Astyanax* linkage groups (LG) 1, 2, 7, 11, 18, and 23. Red line indicates P = 0.05; any value above that threshold is considered statistically significant. Shaded fields indicate the 95% Bayesian confidence interval for the location of each QTL. Colored boxes at the top of the chart indicate the position of eye, lens, retina, or bone QTL found in other studies. [a] = Protas et al. [44], [b] = Protas et al. [45], [c] = O'Quin et al. [29]. Two QTL found here–one for eye area on LG 11 and another for eye diameter on LG 18 –have not previously been described in other studies.
(PDF)

S1 Table. Phenotypic and genotypic data used for QTL analysis of scleral ossification.
(CSV)

S2 Table. Phenotypic and genotypic data used for QTL analysis of eye diameter, eye area, and pupil area.
(CSV)

S3 Table. Summary statistics for all significant QTL found in this study.
(DOCX)

Acknowledgments

We thank two anonymous reviewers for their valuable comments on previous drafts of this manuscript, as well as their suggestions for future work.

Author Contributions

Conceived and designed the experiments: KEO WRJ. Performed the experiments: KEO PD AL EH MY WRJ. Analyzed the data: KEO. Contributed reagents/materials/analysis tools: MY WRJ. Wrote the paper: KEO MY WRJ.

References

1. McBrien NA, Gentle A (2003) Role of the sclera in the development and pathological complications of myopia. Prog Retin Eye Res 22: 307–338. doi: 10.1016/S1350-9462(02)00063-0 PMID: 12852489
2. Franz-Odendaal TA, Vickaryous MK (2006) Skeletal elements in the vertebrate eye and adnexa: morphological and developmental perspectives. Dev Dyn 235: 1244–1255. doi: 10.1002/dvdy.20718 PMID: 16496288
3. Mullaney J, Coffey VP, Fenton M (1971) Atavistic ocular ossicle. Br J Ophthalmol 55: 243–247. PMID: 4995403
4. Pecorella I, Vingolo E, Ciardi A, Grenga P (2006) Scleral ossification in phthisical eyes. Orbit 25: 35–38. doi: 10.1080/01676830500505962 PMID: 16527774
5. Koktekir BE, Karabagli P, Gonul S, Bozkurt B, Gedik S (2014) Extensive bone formation in a painful blind eye. J Craniofac Surg 25: e562–e563. doi: 10.1097/SCS.0000000000001109 PMID: 25364973
6. Hall BK (2005) Bones and Cartilage: Developmental and Evolutionary Skeletal Biology. San Diego: Elsevier Academic Press. 760 p.
7. Thompson H, Griffiths JS, Jeffery G, McGonnell IM (2010) The retinal pigment epithelium of the eye regulates the development of scleral cartilage. Dev Biol 347: 40–52. doi: 10.1016/j.ydbio.2010.08.006 PMID: 20707994
8. Pinto C, Hall B (1991) Toward an understanding of the epithelial requirement for osteogenesis in scleral mesenchyme of the embryonic chick. J Exp Zool 259: 92–108. PMID: 1906525
9. Duench K, Franz-Odendaal TA (2012) BMP and Hedgehog signaling during the development of scleral ossicles. Dev Biol 365: 251–258. doi: 10.1016/j.ydbio.2012.02.016 PMID: 22370003
10. Palmoski MJ, Goetinck PF (1970) An analysis of the development of conjunctival papillae and scleral ossicles in the eye of the scaleless mutant. J Exp Zool 174: 157–164. PMID: 5463648
11. Coulombre JL, Coulombre AJ (1975) Corneal development. V. Treatment of five-day old embryos of domestic fowl with 6-diazo-5-oxo-L-norleucine (DON). Dev Biol 45: 291–303. PMID: 1193300
12. Franz-Odendaal TA (2008) Scleral ossicles of teleostei: Evolutionary and developmental trends. Anat Rec 291: 161–168. doi: 10.1002/ar.20639
13. Kague E, Gallagher M, Burke S, Parsons M, Franz-Odendaal T, et al. (2012) Skeletogenic Fate of Zebrafish Cranial and Trunk Neural Crest. PLoS One 7. doi: 10.1371/journal.pone.0047394
14. Franz-Odendaal TA, Ryan K, Hall BK (2007) Developmental and Morphological Variation in the Teleost Craniofacial Skeleton Reveals an Unusual Mode of Ossification. J Exp Zool Part B-Molecular Dev Evol: 1–13. doi: 10.1002/jez.b
15. Franz-Odendaal TA, Hall BK (2006) Skeletal elements within teleost eyes and a discussion of their homology. J Morphol 267: 1326–1337. doi: 10.1002/jmor.10479 PMID: 17051547
16. Edinger T (1929) Uber knocherne Scleralringe. Zool Jahrbücher 51: 163–226.
17. Jeffery WR, Strickler AG, Yamamoto Y (2003) To see or not to see: evolution of eye degeneration in mexican blind cavefish. Integr Comp Biol 43: 531–541. doi: 10.1093/icb/43.4.531 PMID: 21680461
18. Dufton M, Hall BK, Franz-Odendaal TA (2012) Early lens ablation causes dramatic long-term effects on the shape of bones in the craniofacial skeleton of Astyanax mexicanus. PLoS One 7: e50308. doi: 10.1371/journal.pone.0050308 PMID: 23226260
19. Mitchell RW, Russel WH, Elliot WR (1977) Mexican eyeless characin fishes, Genus Astyanax: Environment, Distribution, and Evolution. Spec Publ Museum Texas Tech Univ 12: 1–89.
20. Yamamoto Y, Espinasa L, Stock DW, Jeffery WR (2003) Development and evolution of craniofacial patterning is mediated by eye-dependent and -independent processes in the cavefish *Astyanax*. Evol Dev 5: 435–446. PMID: 12950623
21. Dufton M, Franz-Odendaal TA (2015) Morphological diversity in the orbital bones of two teleosts with experimental and natural variation in eye size. Dev Dyn. doi: 10.1002/dvdy.24278
22. Wilkens H (1971) Genetic interpretation of regressive evolutionary processes: studies on hybrid eyes of two Astyanax cave populations (Characidae, pisces). Evolution (N Y) 25: 530–544. doi: 10.1554/0014-3820(2002)056[0429:TSFTSO]2.0.CO;2
23. Broman KW, Sen S (2009) A Guide to QTL Mapping with R/qtl. (Statistics for Biology and Health). Springer. 412 p.
24. Protas ME, Hersey C, Kochanek D, Zhou Y, Wilkens H, et al. (2006) Genetic analysis of cavefish reveals molecular convergence in the evolution of albinism. Nat Genet 38: 107–111. doi: 10.1038/ng1700 PMID: 16341223

25. Gross JB, Borowsky R, Tabin CJ (2009) A novel role for Mc1r in the parallel evolution of depigmentation in independent populations of the cavefish Astyanax mexicanus. PLoS Genet 5: e1000326. doi: 10.1371/journal.pgen.1000326 PMID: 19119422

26. Borowsky R, Wilkens H (2002) Mapping a cave fish genome: polygenic systems and regressive evolution. J Hered 93: 19–21. PMID: 12011170

27. Gross JB (2012) The complex origin of Astyanax cavefish. BMC Evol Biol 12: 105. doi: 10.1186/1471-2148-12-105 PMID: 22747496

28. Yoshizawa M, Yamamoto Y, O'Quin KE, Jeffery WR (2012) Evolution of an adaptive behavior and its sensory receptors promotes eye regression in blind cavefish. BMC Biol 10: 108. doi: 10.1186/1741-7007-10-108 PMID: 23270452

29. O'Quin KE, Yoshizawa M, Doshi P, Jeffery WR (2013) Quantitative genetic analysis of retinal degeneration in the blind cavefish Astyanax mexicanus. PLoS One 8: e57281. doi: 10.1371/journal.pone.0057281 PMID: 23437360

30. Hanken J, Wassersug R (1981) The visible skeleton. Funct Photogr 16: 22–26.

31. Franz-Odendaal TA, Ryan K, Hall BK (2007) Developmental and morphological variation in the teleost craniofacial skeleton reveals an unusual mode of ossification. J Exp Zool Part B-Molecular Dev Evol 308B: 709–721.

32. Schneider CA, Rasband WS, Eliceiri KW (2012) NIH Image to ImageJ: 25 years of image analysis. Nat Methods 9: 671–675. doi: 10.1038/nmeth.2089 PMID: 22930834

33. Rasband WS (2014) ImageJ. US Natl Institutes Heal Bethesda, Maryland, USA. Available: http://imagej.nih.gov/ij.

34. Pagel M (1999) The maximum likelihood approach to reconstructing ancestral character states of discrete characters on phylogenies. Syst Biol 48: 612–622. Available: http://sysbio.oxfordjournals.org/content/48/3/612.full.pdf.

35. Coghill LM, Darrin Hulsey C, Chaves-Campos J, García de Leon FJ, Johnson SG (2014) Next generation phylogeography of cave and surface Astyanax mexicanus. Mol Phylogenet Evol 79C: 368–374. doi: 10.1016/j.ympev.2014.06.029

36. Dowling TE, Martasian DP, Jeffery WR (2002) Evidence for multiple genetic forms with similar eyeless phenotypes in the blind cavefish, Astyanax mexicanus. Mol Biol Evol 19: 446–455. PMID: 11919286

37. Strecker U, Bernatchez L, Wilkens H (2003) Genetic divergence between cave and surface populations of Astyanax in Mexico (Characidae, Teleostei). Mol Ecol 12: 699–710. PMID: 12675825

38. Mok H, Liu S-H (2012) Morphological variations in the scleral ossicles of 172 families of Actinopterygian fishes with noes on their phylogenetic implications. Zool Stud 51: 1490–1502.

39. Maddison WP, Maddison DR (2014) Mesquite: A modual system for evolutionary analysis. Version 2.75. Available: http://mesquiteproject.org.

40. Manichaikul A, Moon JY, Sen S, Yandell BS, Broman KW (2009) A model selection approach for the identification of quantitative trait loci in experimental crosses, allowing epistasis. Genetics 181: 1077–1086. doi: 10.1534/genetics.108.094565 PMID: 19104078

41. Borowsky R (2008) Restoring sight in blind cavefish. Curr Biol 18: R23–R24. doi: 10.1016/j.cub.2007.11.023 PMID: 18177707

42. Wright S (1934) The results of crosses between inbred strains of guinea pigs, differing in number of digits. Genetics 19: 537–551. PMID: 17246736

43. Lynch M, Walsh B (1998) Genetics and analysis of quantitative traits.

44. Protas M, Conrad M, Gross JB, Tabin C, Borowsky R (2007) Regressive evolution in the Mexican cave tetra, Astyanax mexicanus. Curr Biol 17: 452–454. doi: 10.1016/j.cub.2007.01.051 PMID: 17306543

45. Protas M, Tabansky I, Conrad M, Gross JB, Vidal O, et al. (2008) Multi-trait evolution in a cave fish, Astyanax mexicanus. Evol Dev 10: 196–209. doi: 10.1111/j.1525-142X.2008.00227.x PMID: 18315813

46. Espinasa L, Rivas-Manzano P, Perez HE (2001) A new blind cave fish population of genus Astyanax: geography, morphology and behavior. Environ Biol Fishes 62: 339–344.

47. Wilkens H, Strecker U (2003) Convergent evolution of the cavefish Astyanax (Characidae, Teleostei): genetic evidence from reduced eye-size and pigmentation. Biol J Linn Soc 80: 545–554.

48. Duboué ER, Keene AC, Borowsky RL (2011) Evolutionary convergence on sleep loss in cavefish populations. Curr Biol 21: 671–676. doi: 10.1016/j.cub.2011.03.020 PMID: 21474315

49. Beale A, Guibal C, Tamai TK, Klotz L, Cowen S, et al. (2013) Circadian rhythms in Mexican blind cavefish Astyanax mexicanus in the lab and in the field. Nat Commun 4: 2769. doi: 10.1038/ncomms3769 PMID: 24225650

50. Elipot Y, Hinaux H, Callebert J, Launay J-M, Blin M, et al. (2014) A mutation in the enzyme monoamine oxidase explains part of the Astyanax cavefish behavioural syndrome. Nat Commun 5: 3647. doi: 10.1038/ncomms4647 PMID: 24717983

51. Langecker TG, Longley G (1993) Morphological adaptations of the Texas blind catfishes *Trogloglanis pattersoni* and *Satan eurystomus* (Siluriformes: Ictaluridae) to their underground environment. Am Soc Icthyologists Herpetol 1993: 976–986.

52. Schobert CS, Stiassny MLJ, Schwab IR, Zeiss C, Schelly RC, et al. (2013) Comparative ocular anatomy in a blind African cichlid fish, Lamprologus lethops. Vet Ophthalmol 16: 359–364. doi: 10.1111/vop.12006 PMID: 23186233

53. Poulson TL (2001) Adaptations of cave fishes with some comparisons to deep-sea fishes. Environ Biol Fishes 62: 345–364.

54. Wilkens H (1988) Evolution and genetics of epigean and cave Astyanax fasciatus (Characidae, Pisces): In Support for the Neutral Mutation Theory. In: Hecht M, Wallace B, editors. Evolutionary Biology, Vol 23. New York. pp. 271–367.
